# Role of Water on the Rotational Dynamics of the Organic Methylammonium Cation: A First Principles Analysis

**DOI:** 10.1038/s41598-018-36900-4

**Published:** 2019-01-24

**Authors:** Ross D. Hoehn, Joseph S. Francisco, Sabre Kais, Ali Kachmar

**Affiliations:** 1 Department of Chemistry, Department of Physics and Birck Nanotechnology Center, Purdue University, West Lafayette, Indiana, 47907 USA; 2 Qatar Environment and Energy Research Institute, Hamad Bin Khalifa University, Qatar Foundation, P.O. Box 5825, Doha, Qatar; 3 Department of Chemistry, University of Nebraska-Lincoln, Lincoln, Nebraska 68588 USA

## Abstract

Understanding the degradation mechanisms of lead-halide perovskites (CH_3_NH_3_PbI_3_) under exposure to liquid/aerosol water is an essential problem within the photovoltaic community. Herein we investigate both the static and the dynamic properties of the methylammonuim cation (MA) as it coordinates with invading water molecules (MA.(H_2_O)_*n*_, *n* = 1, 2, 3, 4) using both stationary state quantum mechanics and first principle molecular dynamics simulations. Various solvation structures of MA were characterized by their stabilization energies, dipoles, and Maximally-Localized Wannier Function (MLWF) centers. Calculation – and analysis – of vibrational shifts in the IR spectral region were performed for hydrated complexes; the locations of ${\bf{N}}{{\bf{H}}}_{{\bf{3}}}^{{\boldsymbol{+}}}$ stretching vibrations allude to significant hydrogen bonding between MA and the water molecules. Through Fourier analysis of the rotational dynamics on several MA · (H_2_O)*n* complexes, we conclude that the water molecules dampen the rotational motion of the MA as the intermolecular bonds formed between the water molecules and the MA act to hinder the rotation of the cation; these findings give explanatory support to earlier computational observations of humidity effects on perovskites (i.e., CH_3_NH_3_PbI_3_) materials. This work is a step toward understanding the water-MA cation interaction in bulk perovskites, thus providing greater understanding of *in situ* instability/degradation of perovskite bulk materials.

## Introduction

Lead halide perovskites such as CH_3_NH_3_PbI_3_ (MAPbI_3_) (MA $\equiv$ Methyl-ammonium) are key candidate materials for energy conversion from light to electricity^1^. However, these materials suffer from instabilities owing to decomposition upon exposure to water vapor, humid air and ultraviolet (UV) radiation^2–4^. The crystal structure of lead halide perovskites (MAPbI_3_) can be seen as the deposition product between the two charged species: (PbI_3_)^−^ and MA^+^ (CH_3_NH_3_)^+^^5–7^. It has been suggested that MAPbI_3_ decomposes into PbI_2_ and MAI; where MAI further dissociates into iodine, hydrogen, HI and water^8^. This decomposition reaction is considered to be proceeded by a strong interaction between H_2_O and MA via an ${H}_{2}O\cdots H-N$ intermolecular bond in CH_3_NH_3_PbI_3_ + (H_2_O)*n* (*n* is the number of water molecules), which leads to a decomposition of the [PbI_3_]^−^ cage containing the [CH_3_NH_3_]^+^ cation. However, the degradation mechanism of MAPbI_3_ is not yet fully understood.

Recently, both experimental and theoretical efforts have been focused both on examining and on proposing degradation mechanisms for lead halide perovskites under either UV exposure or humidity^9^. Frost *et al*. proposed a decomposition pathway for MAPbI_3_ considering the aqueous solubilities of MA and HI^10^, yet lead halide is insoluble^11^, while Qiu *et al*. proposed that the degradation progressed in two steps. Firstly, CH_3_NH_3_PbI_3_ decomposes into aqueous CH_3_NH_3_I and PbI_2_ in the presence of H_2_O. CH_3_NH_3_I then decomposes into CH_3_NH_2_ and HI, where HI may react with O_2_ or may photodecompose into H_2_ and I_2_ ^6^. Improved photoluminescence of lead halide perovskite film after having been exposed to moisture has been reported; this improvement could be due to partial solvation of–or coordination of water with–the methyl-ammonium component^11^. Humidity effects on the perovskite materials suggest that infiltrating waters interact with the MA within the lead halid cage, as well as with the iodide lattice. Once the water infiltrates into the bulk-phase perovskite, it forms a hydrate phase with either the local iodide or MA; in some capacity, this hydration is responsible for the decay of the charge carrier capacity within the material^12,13^. Investigations of water’s interaction with both PbI_2_ and MA $\cdot$ I terminated perovskite surfaces. It was observed that water penetrated into the perovskite lattice at PbI_2_-terminated surfaces, whereas the MA $\cdot$ I evacuated to the MA $\cdot$ I-terminated surface^12^. Leguy *et al*. reported that water can have either a positive or a negative impact on the charge transport performance of the perovskite cell devices^14^. Using non-adiabatic *ab initio* molecular dynamics, Lung *et al*. reported that moderate humidity could delay electron-hole recombination in hybrid organic perovskites^15^, this is possibly due to water playing a role in shifting and altering some vibrational modes of the MA. This finding is therefore likely associated with the hydrogen bonding interaction between H_2_O and MA, which is stronger than the hydrogen bonding between MA and the iodide lattice sites (PbI_3_)^6^. This is typically possible only for low concentrations of liquid water, where one may achieve a reversible aqueous phase of MAPbI_3_ ^14^.

Within CH_3_NH_3_PbI_3_ perovskite, MA is known to rotate quickly with a relaxation time of 5.47 ps at 268 K^16^, a barrier height for rotation from (011) to (111) direction of 20 meV^17^, an energetic barrier of rotation is 13.5 meV^18^, and a free energy barrier between favorable orientations is 12.5 meV^19^. To more deeply address the role of water on the rotational dynamics of MA, and to draw possible solvation scenarios, likely responsible for the strong electron-phonon coupling in perovskites, we investigate water’s interaction with a free Methyl-ammonium cation. The interaction between the MA and each water molecule is electrostatic in nature $[({{\rm{NH}}}_{3}{)}^{\delta +}\cdots {O}^{\delta -}]$, forming a consistent hydrogen bond between the ammonium’s nitrogen and the oxygen of a water molecule. However, water can also exhibit strong intermolecular bonding with the surrounding lattice, mostly with the iodide. These water-based hydrogen bonding interactions provide anchoring points on both the rotating organic and inorganic cage, facilitating a hindered rotation of the organic within the perovskite lattice.

In this work, both static Density Functional Theory and *ab initio* molecular dynamics simulations are applied to investigate the MA’s rotation, dipole probability distributions, the interactions between water molecules and an MA considering different conformational isomers. Both static and dynamic simulations are used to analyze the energetics of both the degradation and solvation processes. According to our calculations, MA is prone to solvation, a process characteristic of strong interactions between the amine and the oxygen of the water molecules. The remains of this paper are organized as follows: $\S$2 conveys the theoretical formalities used within this paper; $\S$3.0.2 and 3.0.3 contains information concerning the statics of the hydrated complexes, including stabilization energies, charges and spectroscopic analysis; $\S$3.1 conveys an analysis of the system dynamics, including isomeric structures, hydrogen bonding and Fourier analysis; finally, $\S$4 provides closing statements.

## Computational Details

Molecular dynamics simulations were performed using the CP2K code^20^; additionally all the geometry optimizations were carried out on all configurations for the MA $\cdot$ (H_2_O)_*n*_ hydrated complex using CP2K^20–29^. The Wavelet Poisson Solver was employed to obtain a good description of the molecular wave function in non-periodic degrees of freedom, permitting the removal of spurious interactions within a system possessing its own periodic images. We performed molecular dynamic simulations on the optimized structure of each isomer, also using the CP2K code. Gaussian and Plane Waves methods (GPW) implemented through the Quickstep module of the CP2K package were employed. Temperature control was implemented for the ionic degrees of freedom by using Nosé-Hoover thermostats with three chains, under a target temperature of 300 K and with a time constant of 50 fs. The time step during the integration of the dynamic equations was set to 0.5 fs. Electronic structure properties were calculated using the PBE^29^ and BLYP^30,31^ functionals with the empirical Grimme correction scheme (DFT-D3) to account for the dispersion interactions^32,33^, which are vital in the present system. Kohn-Sham orbitals are expanded in a Gaussian basis set for the all atoms (MOLOPT-DZVP-SR-GTH for C, N, O, H), and we employed the norm-conserving GTH pseudopotentials. An energy cutoff of 550 Ry and a relative cutoff of 70 Ry were employed for the auxiliary plane wave (PW) basis set. Periodic Boundary Conditions (PBC) were applied in the *ab initio* MD simulation environment. The simulation was allowed to evolve to 42 ps of simulation time. Static electronic structure calculations were carried out using the Gaussian 09 suite with the PBE, and wb97xd functionals with the aug-cc-pvtz basis set applied to compute the energies, natural bonding orbital (NBO) and Mulliken charges, which are provided in the SI (Tables II, III)^34^. The IR spectra for the (MA.(H_2_O)_*n*_, *n* = 1, 2, 3, 4) complexes were computed using static method (diagonalization of the dynamical matrix) as implemented in CP2K. Dipoles were collected on the fly subjected to a localization procedure using the MWLF approximation^35–37^. Discrete Fourier Transform (dFT) analysis were evaluated using the mathematical Nyquist-Shannon sampling theorem to determine qualitative alterations in the rotational dynamics.

## Results and Discussion

### Isomers stabilization energies

Firstly, we shall discuss the stability of the MA with respect to the solvation of water molecules. We initially optimize the structure of each solvated MA isomer with *n* number of water molecules, where *n* ranges from 1 to 4. Figure SI1 displays the optimized geometries of several MA $\cdot$ (H_2_O)*n* complexes. Stabilization energies for these complexes were determined by comparison of the optimized complex with individual optimizations of each molecular component and evaluation of total energy carried out on independent water molecules (${{\rm{E}}}_{{H}_{2}O}$), water clusters (${{\rm{E}}}_{{({H}_{2}O)}_{n}}$) and MA (E_*MA*_). The stabilization energy is computed with respect to the sum of the fragment energies, in a fashion similar to the binding energy ΔE:

1 $${\rm{\Delta }}E={E}_{MA\cdot {({H}_{2}O)}_{n}}-{E}_{{({H}_{2}O)}_{n}}-{E}_{MA},$$

where ${E}_{MA\cdot {({H}_{2}O)}_{n}}$ is the total energy of the *n*-water hydrated complex, E(H2O)*n* is the total energy of the water cluster (monomer, dimer, and trimmer), and E(MA) is the total energy of a free MA. The results of the stabilization energy are given in the Table SII. It is worth noting that the MA commits rotational motions, which involve the displacement of the water molecules solvating the amine. Following the rotation, the MA $\cdot$ (H_2_O)*n* configurations will form dimers, trimers, tetramers and lone water molecule clusters within the complex; thus we re-compute these stabilization energies with respect to the appropriate water clusters in their optimized geometries. It can be noted from Table SII that the stabilization energies for each of the various hydrated complexes under present consideration increase with respect to the number of waters. This suggests that the stabilization of MA is associated with the strength and the number of water hydrogen bond network. However, the lowest stabilization of any of the hydrated complexes belongs to two conformers (Isomer 1, Isomer 2) of the *n* = 4 case, see Fig. SI1(g,h).

### Spectroscopy analysis

Spectroscopy analysis are very important to characterize the hydrogen bonding networks in water and for hydrated cations in water. These kind of analysis will allow us to characterize the hydrogen bonds between the (NH_3_)^+^ group of the MA and the water molecules, and also to capture the changes of N-H stretch vibration that are known for their sensitivity to the strength of hydrogen bond network.

**Figure 1:**
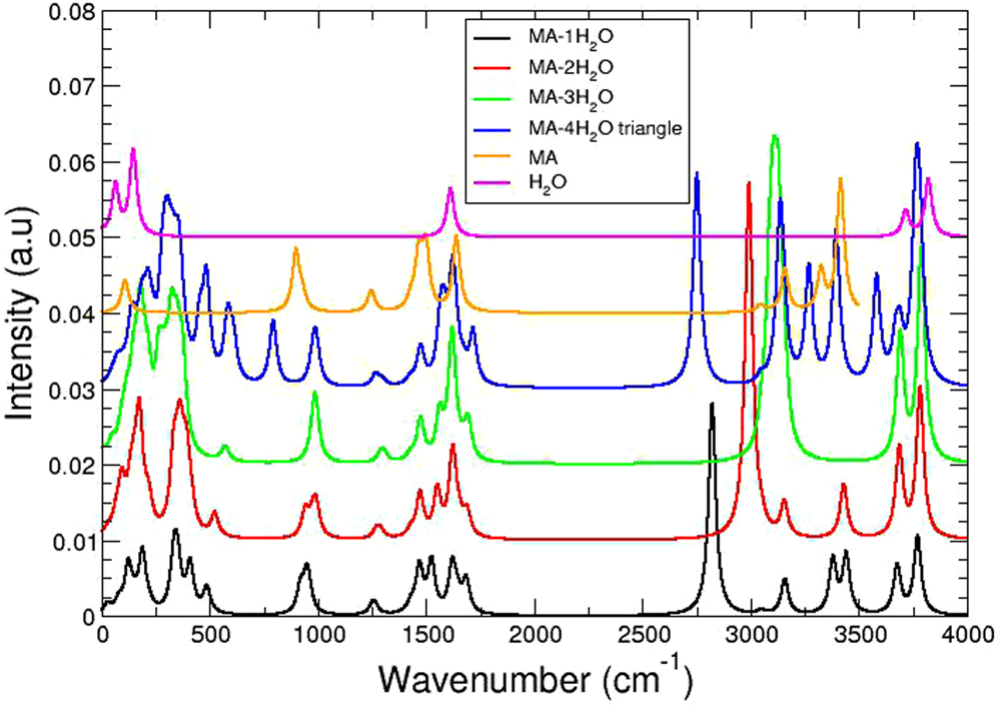
IR spectra of MA.n(H_2_O) (1 = < n < = 4). Starting from the bottom, successive data set pairs are shifted up by 0.025 units.

To characterize the effects of hydrogen bonding between the water molecules and the MA, we calculate the IR spectra for each MA $\cdot$ (H_2_O)_*n*_ complex, determining energy shifts in vibrational modes due to solvation. We show in Fig. 1 the vibrational spectrum for various isomers of the MA $\cdot$ (H_2_O)_*n*_ hydrated complexes, namely the isomers given as subfigures (a), (b), (d), (g) in Fig. SI1, as well as the spectra of both MA and H_2_O. All the relevant bands appeared to be well pronounced in the computed spectra. The IR intensities for the particular modes discussed below are listed in Table SIIV with their spectral assignments and frequencies. From the IR spectra, we find that there are roughly two regions which undergo pronounced alterations in their modes/frequencies during solvation and are separated by a small mid-energy region featuring stable characteristics. The first region lays in the lower energy domain, below ~900 cm^−1^, where novel modes come about due to complex, collective intermolecular motions. An example of these modes would be the collective hindered rotations of h-bonding species in the realm of 500–850 cm^−1^ for all hydrated complexes, with pronounced subsequent peaks in the *n* = 4 case. Within the stable separation region, 900–1485 cm^−1^, a number of modes do not directly interplay with the intermolecular bonds; examples being: N-C stretch and methyl bending modes.

In the higher frequency region, several atoms participate in modes directly involved in the intermolecular forces forming the hydrated complexes. The region of ~1500–1750 cm^−1^ is home to the bending modes of the *n* waters and the NH_3_ bending modes; due to the proximity of these bands to each other–with the subsequent addition of more waters–their motions begin to couple, forming new bands which no longer strictly conform to their original spectroscopic assignments. These absorption bands are effectively blue shifted–as the optimized intermolecular structure sits at an energetic low point in conformation space–as they are the cause of geometric deformations from the optimized geometry. Additionally within this higher energy region, the CH_3_ stretching modes have nearly no effect on the intermolecular bonding and display only slight shifts, yet are effected by the breaking of symmetry caused by NH_3_-associated waters in several hydrated complexes. The NH_3_ stretching modes are capable of undergoing severe red shifting in cases where the hydrogen’s vibratory motion is eased by the presence of another partially negatively charged atom (oxygen) when the vibratory motion is along an h-bond. (For verification, both the Muliken and NBO charges for all atoms within the hydrated complexes are given in Tables SIII and III) The high energy vibrations belonging to the water’s stretching modes are capable of collectivizing their motions (minor red shifting), and being effected by h-bonding vectors (severe red shifting). All relevant computed vibrations of each of the hydrated complexes are in good agreement with reported data concerning invading waters in perovskite lattice structures^13^.

### Wannier Dipoles

Since both MA and water molecules are polar with 2.6 D^36^ for water and 2.2 D for the MA^38^. Therefore it is of our interest in this study to compute over the molecular dynamics simulations the total dipole moment of the different hydrated complexes to understand the changes of the dipole probability, the interaction between the MA and the water molecules, and the MA rotational behavior.

**Figure 2:**
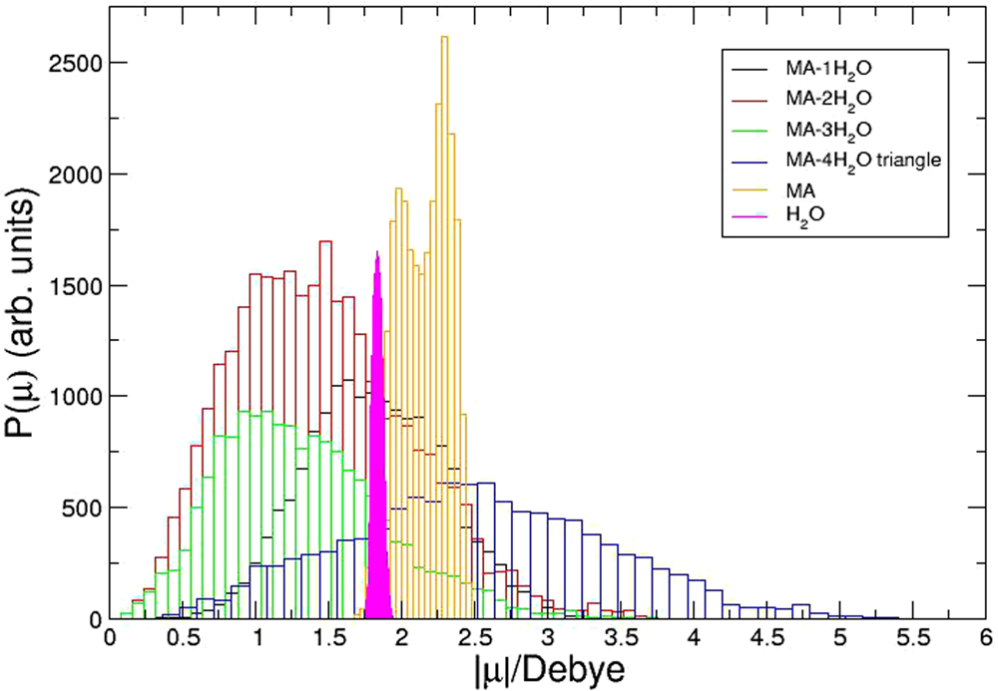
Dipole probabilities for MA.n(H_2_O) (1 < = n < = 4), MA, and H_2_O.

As the interactions between the MA and each water molecule are effectively charge-charge and dipole-dipole in nature, we computed the total dipole moment of each isomer discussed above. Starting from the optimized structures of the isomers (a), (b), (d) and (g) within Fig. SI1, we performed NVT MD simulations lasting 42 ps during which the Berry phase method was employed to compute the total dipole moment at each time step. In order to study the dipolar properties of the system the electron density was subjected to an on-the-fly localization procedure using Maximally Localized Wanniers Functions (MWLF)^35–37,39–41^. The Wannier Centers analysis are regarded as centers of electron pair charge density in local orbitals, and with this approach the individual dipole moment of the hydrated complex and each constituent species can be computed. A population density histogram for the total dipole moment is given as Fig. 2.

From Fig. 2, it is clear that the dipole of water is recovered quite well. The bifurcation of MA’s dipole population is likely an artifact of low sampling density, while the average of the total population does agree with the static quantum mechanical value. It appears very difficult to quantify the average dipole moment for the various MA $\cdot$ (H_2_O)_*n*_ isomers from the histograms, considering the collective dynamics associated with the solvation of MA abide a Boltzmann-like distribution. It should be noted that the population maximum of each histograms do conform to logical predictions. The weakest of the dipoles was easily predicted to be the *n* = 3 case, where the water molecules form a trimeric shape and their in-plane dipole components nearly cancel, while their vertical component is in geometric opposition to the MA dipole. The strongest dipole strength at the population maximum is the *n* = 4 case, as the orientations of the waters within the triangular structure no longer cancel in vector addition due to the fourth water. The *n* = 1 case has one water, whose in-plane dipole component cancels with nothing, and whose vertical component (being a single water) has only slight cancellation with the MA; the *n* = 2 falls into place, slightly south of middle.

Generally, changes associated with the dipole probability are important observables for both the MA rotation and for water displacement during MA’s rotation about the CN axis. The dipole probability result suggest that Raman could be a great tool to measure the relative humidity within perovskite cells.

### Structure and dynamic properties

**Figure 3:**
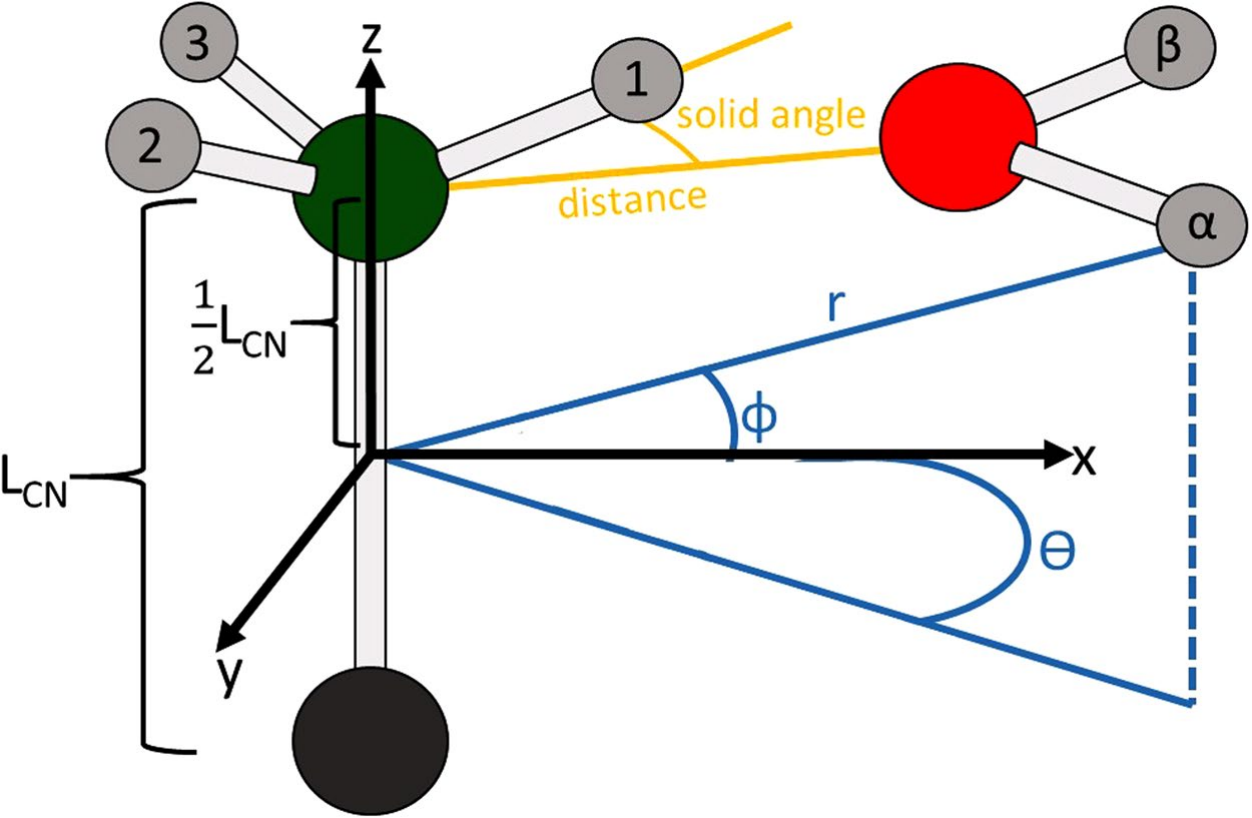
Schematic representation of the MA-water interaction coordinate systems. Within the plot, *L*_*CN*_ is the length of the nitrogen-oxygen bond; *x*, *y*, *z* are the standard Cartesian coordinates; and *r*, *ϕ*, *θ* are the polar coordinate system (following standard mathematical). The pertinent h-bonding coordinates (donor-donor distance and donor-acceptor-donor solid angle) or H1–N–O are given in yellow.

The focus of this section is on the dynamics of water molecules associated with MA. A schematic representation of a single water interacting with primary ammonium terminus of MA amine is given as Fig. 3. To track the relative positions and orientations of each atom within the hydrated system a polar coordinate system is employed; this coordinate system–and the respective Cartesian system–are both described within Fig. 3. The carbon-nitrogen bond is initially aligned with the *z*^*th*^ axis of the Cartesian (and polar) coordinate system(s) with the system origin consistently placed at the midpoint nitrogen and carbon bond (*L*_*CN*_), initially placing nitrogen at $z=+\,\frac{1}{2}{L}_{CN}$. At t = 0 of the dynamics, an arbitrary ammonium hydrogen is selected (henceforth known as Hydrogen 1, or H1) and aligned with the *x*-axis of a Cartesian coordinate system; the remaining ammonium hydrogens are canonically labeled–Hydrogen 2 (H2) and Hydrogen 3 (H3)–and tracked consistently within the simulation. All *n* waters within a particular simulation are labeled and consistently tracked. During the dynamics, the polar and azimuthal angles (*ϕ* and *θ*) of all atoms are recorded. Furthermore, the distance between h-bond donor sites–nitrogen and the oxygen of each water–and the solid angle of each possible h-bonding donor-acceptor-donor arrangement are calculated.

**Figure 4:**
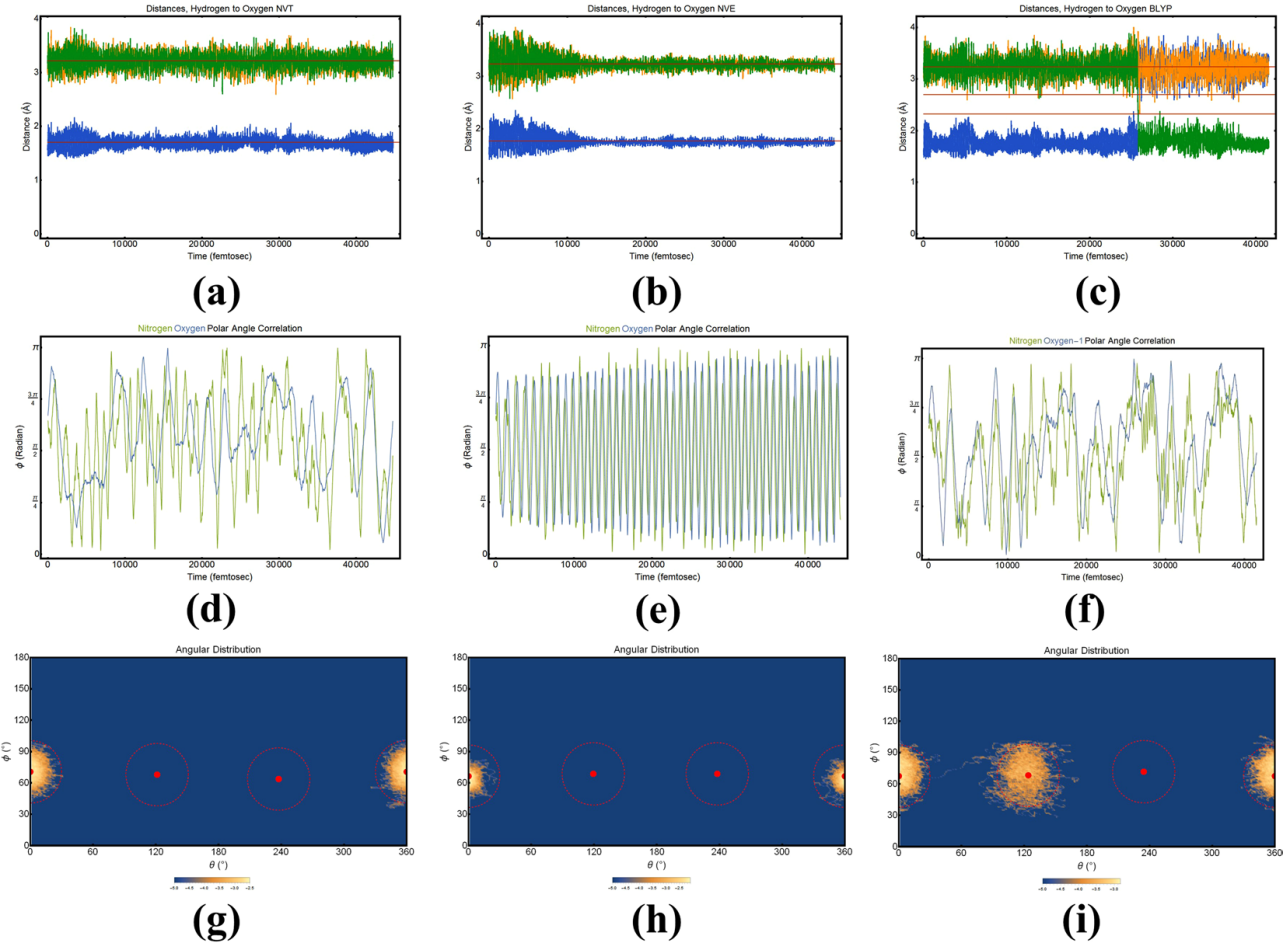
Column 1–3 are representative of calculations using NVT(PBE), NVE(PBE) and NVE(BLYP) methods. Row 1 are the Hydrogen-Oxygen distance in the hydrogen bonding complex. Row 2 gives the time dynamics of the polar angular coordinate. Row 3 gives the angular distributions of the water’s oxygens about each of the hydrogen of MA.

Within the subsequent sections we will describe the dynamic behaviors of several hydrated complexes conforming to the formula MA $\cdot n$ H_2_O, where *n* = 1, 2, 3, 4. As we are predominately concerned with the dynamic behavior of the h-bonding interactions and collective rotations, within each section we will employ a selection of four types of plots. These plots will be outlined here for ease of future reading. 1. H-bond distance plots (*e.g*. Fig. 4(a–c)) gives the distance in angstrom (Å) between each labeled ammonium hydrogen (designated through color) and the oxygen of a single (of *n*) water molecule. The average distances are given as red lines. This plot both permits the evaluation of h-bond formation through the distance and allows us to observe the exchange of a water molecule within the hydrated complex. 2. Angular probability distribution plots (*e.g*. Fig. 4(g,h) give the population density (log-scaled for readability) of oxygen atoms (collective for all of *n* waters) with respect to each of the hydrogen (shown as a dot surrounded by a circle denoting a solid angle of 30 deg, defined as a limiting angle to h-bonding). Such a plot, again, allows us to appraise the quality of h-bonding in the system. 3. Time dependent angular trajectory plots (*e.g*. Fig. 4(d–f)) follow the evolution of *ϕ* (*ϕ* plots is given in the main paper, *θ* plots are given within the SI) during the simulation. These plots will contain the time-evolution of the polar coordinate for ammonium nitrogen and each of the relevant water oxygens. Such a plot displays the development of collective rotations (as h-bond formation associates a water to a ammonium hydrogen); these collective rotations have consequences for the rotational dynamics of the MA which (we believe) would be exacerbated if we consider the perovskite cage, and the water’s interactions with both the cage and the MA would more closely mimic an asymmetric hindered rotor. 4. Cloud plots (*e.g*. Fig. SI6(a,b)) are the graphical representation of the distribution of waters about the MA molecule where **(a)** is colored coded to follow specific waters and **(b)** is encoded to describe the h-bonding (green being a formed h-bond, yellow meaning that it either conforms to the distance or angle portions of the definition but not both, and red being no observed h-bond).

#### MA $\cdot$ H_2_O

We initially chose to perform MD simulations under both the NVT and the NVE canonical ensembles, as well as an NVE MD simulation using a DFT-BLYP functional (see $\S$2). To characterize the intermolecular bonding between the MA and the oxygen of the water molecule, we first analyze the relevant distances between each of the amine hydrogen–H*i*, where *i* = *α*, *β*, *γ*–and the oxygen atom of the water throughout the simulation trajectory. The distances between each the amine hydrogen and the oxygen, $[({{\rm{NH}}}_{3}{)}^{\delta +}\cdots {O}^{\delta -}]$, within the different canonical ensembles are given in the first row of Fig. 4; the subfigures (a), (b) and (c) give the distances for MD simulations under the NVT, NVE and NVE-BLYP methods, respectively. Recalling that the hydrogen’s are consistently labeled within the plots and are designated by color (green, blue, and orange). The later are coded and tracked consistently between the plots. The primary (most proximate) distance within each subfigure is ~1.6 Å, and is in the typical range of the respective hydrogen bond. The secondary distances are longer at ~3.0 Å. The short primary intermolecular bond between a hydrogen and the oxygen, at ~1.6 Angstrom, is suggestive a rather strong interaction.

It is clear from the first row of Fig. 4(a,c) that the strong intermolecular bonding within the two canonical ensembles (NVT, NVE) employing the PBE functional display a single hydrogen association (we will reserve the term h-bond until an evaluation of both the distance and angle) is maintained during the length of the simulation, surviving throughout all inter- and intra- molecular motions. Within Fig. 4(b), a strengthening of the association between the hydrogen and oxygen is observed through the seemingly new equilibrium distance at roughly 13,000 fs. This continuously maintained hydrogen association is not displayed within the NVE-BYLP simulation, noting that the water’s oxygen transition between amine hydrogens at roughly 26,000 fs.

Although one simulation method revealed a migration from one amine hydrogen to another, whereas the other two methods do not, a single hydrogen association is nearly ubiquitously maintained throughout the series of simulations despite the intra-molecular motions of the species. This stable intermolecular bonding is evidenced in row two of Fig. 4 where we show the time evolution of the polar coordinate (at fixed radial coordinate) of both the amine nitrogen (*ϕ*_*N*_) and the water oxygen (*ϕ*_*O*_); as can be seen sub-figures (d)–(f), the fast rotational evolution of *ϕ*_*N*_ is closely shadowed by the slower evolution of the water. The relative speeds do require a minute explanation, as MA is heavier than water, the MA should be slower to rotate; this fast-MA-Slow-water paradigm is due to the definition of the internal coordinates previously discussed; these coordinates effectively treat MA as if it were undergoing a rotational mode, while water is tracking this rotational mode by traversing the conformational space of intermolecular arrangement. Figure SI4 gives a similar series of plots, but for the azimuthal angle; these plots show a similar shadowing of the ammonium by the water oxygen as seen in the polar angle. Figure SI4(a,b) gives the polar and azimuthal coordinates for both the MA carbon and nitrogen, respectively. This is given for ease of understanding the Fourier analysis; the discussion of these plots, and the such plots for other isomers will be reserved for $\S$3.1.3 Furthermore, Fig. SI3 give the polar and azimuthal evolution of the *n* = 0 simulation. The behavior of the system is periodic in both angular variables, with a rotational period of the polar coordinate is roughly 1000 fs, Such a periodic behavior is to be expected as the rotations have no external bias or frame of reference.

A complete statement concerning the nature of the intermolecular bonding requires an analysis of the solid angles between the candidate h-bonding donor-acceptor pairs; this solid angle is visually represented within the third row of Fig. 4. It is, again, evident that strong intermolecular interactions are holding these two chemical species together; this intermolecular interaction is highly localized within 30° (represented by the dotted circles) of the hydrogen-nitrogen occultation (solid dot). Because of both the tight range of distances and the compact distribution of solid angles, we can conclusively state that the strong intermolecular interaction involved in this system are indeed h-bonds. It is also notable that the aforementioned (in the distance plots) exchange of h-bond donor sites within the ammonium in the NVE-BLYP simulation, see Fig. 4i. A graphical representation of the h-bonds within the single water hydrated complex can be found in Fig. SI6; subfigure (a) shows that a single oxygen (represented as a blue cloud projected on a unit sphere) maintains it’s association with a h-bond donor location on ammonium, different specific oxygens will be represented by different colors. In Fig. SI6(b), the water’s oxygen positions are given projected onto a unit sphere. Within this plot the persistent green coloration of the oxygen’s distribution is due to a persistent h-bond being formed, whereas orange would represent an appropriate distance alone (not angle) and red would represent no h-bond based on both a failure in both distance and solid angle criteria.

The intent of this section was three-fold: First, we were required to verify that the strong intermolecular interactions binding the collective rotation of the hydrated complexes are–in fact–h-bonds. Secondly, we introduce the reader to both the concept and reading of the various plots used within this work. Finally, it is necessary to introduce a point-of-reference to the reader for the remaining findings concerning the remaining hydrated complexes. The MD simulation results discussed in the subsequent sections were all performed under NVT conditions, using the PBE functional to reduce the molecular dynamics computational cost as BLYP is more expensive. We will focus our discussion on the log bivariate probability distribution plots as they contain a majority of the information required for the discussion, while other plots to be mentioned shall be given in the SI.

#### MA $\cdot$ 2H_2_O

**Figure 5:**
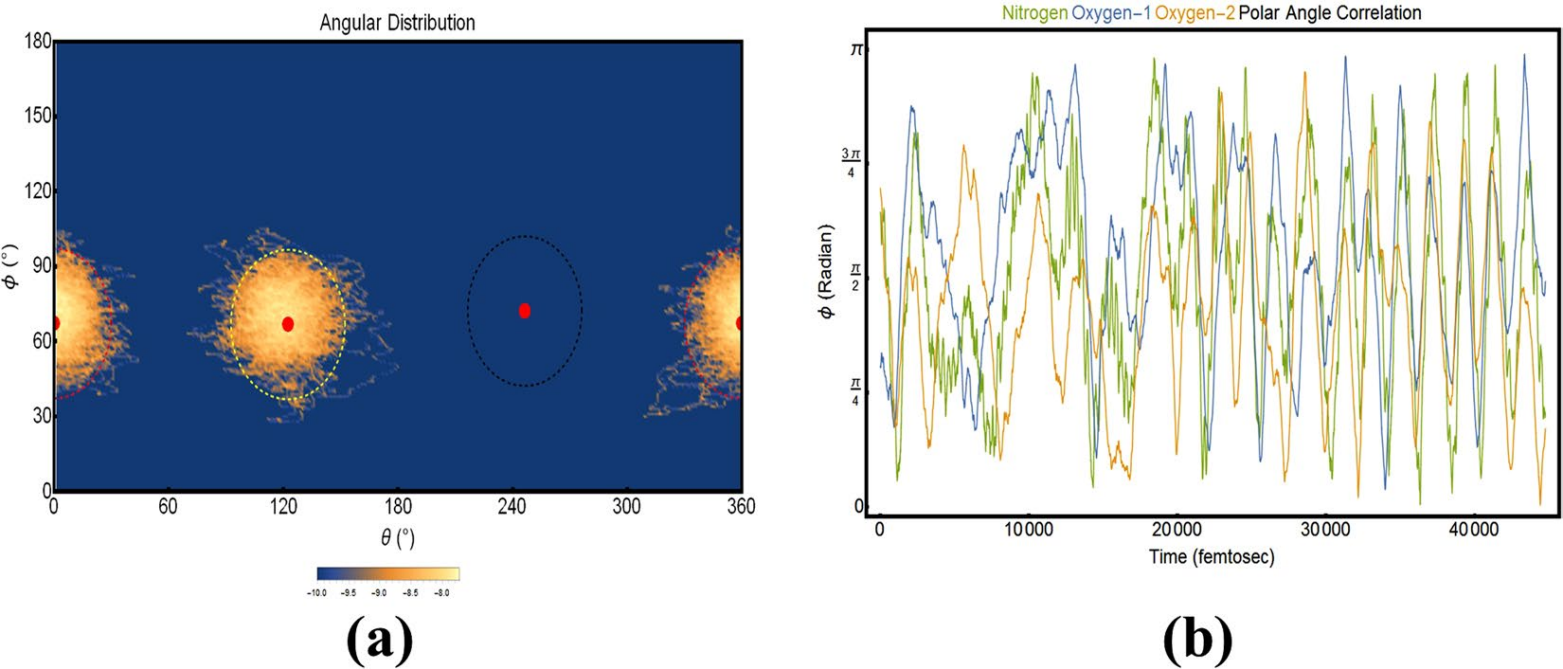
Informations regarding the dynamics for the 2 water case. (**a**) Gives the angular distributions of the water’s oxygens about each of the hydrogen of MA. (**b**) Gives the time dynamics of the polar angular coordinate.

Moving on preliminarily noted that this complex mayto the *n* = 2 complex, it should be take two isomeric forms. The first isomer is represented by the MA interacting with each of the two water molecules via individual h-bonds. The second isomer is represented by a h-bonding interaction between the ammonium and a single water, whereas the second water forms a hydrogen bonded bridge with the first water; hereon, we may refer to such a water as dangling (or a dangler). It is evident from Fig. 5(a) that each of the two waters display a high degree of localization to a specific and separate h-bond donning site on MA. This high locality with only minor walks in confirmation space–taken with the roughly stable association length shown in Fig. SI7(c,d) –imply that the isomer with a dangling water does not occur within this simulation for any appreciable length of time. This locality is evident in the cloud plots, Fig. SI10(a,b). From the distance plots, it should be noted that there exists a paradigm shift in the variance of time-dependent association distance values; this transition occurs at roughly 24,000 fs where the associations appear to get slightly stronger. This paradigm change is also evident in the C-N polar and azimuthal coordinates, Fig. SI8(a,b), respectively. Figure 5b shows the close shadowing behavior evident with each water and its h-bonding site along the polar coordinate; this is reiterated with the azimuthal coordinate in Fig. SI9(a,b) for the individual waters, and (c) all shown together. An analysis for the water-water interactions was performed and is relegated to the SI, see Fig. SI11.

Discussion of complexes displaying greater numbers of water are reserved for the SI. See SI $\S$II.D and II. E.

#### Fourier analysis

To determine the rotational characteristics of the MA as it forms hydrated complexes we must determine the rotational frequencies of the MA (and waters) with respect to each angular degree of freedom. This is done by performing a discrete Fourier analysis on the dynamic for the polar, *ϕ*, and azimuthal, *θ*, degrees of freedom. After performing a discrete Fourier Transform (dFT), we remove redundant information arising from the dFT’s conjugate symmetry. If the entire sampling domain of a typical MD run is accounted for in the dFT, the Nyquist-Shannon sampling theorem suggests that the lowest frequency resolvable is ${f}_{max}=\frac{1}{2}{f}_{s}=1.251$ PHz. The resultant data can be collected and tabulated–see Tables SIV–VII for a list of the important contributing frequencies–or through graphical means, see Figs SI25–29.

Within the *n* = 0 case, we find no justification for cleaving the data domain into subdomains prior to applying a discrete Fourier transform (dFT), due to the regularity of the dynamics. We we suggest that this regular, isolated rotation is akin to the natural rotational frequency of the MA itself, at $\nu  \sim 0.001117$ PHz = 1.117 THz. This should be compared to the quantum mechanically determined rotational period of a typical diatomic molecule, such as CO at ~0.7 THz in vacuum. While this rotational frequency is unlikely to be the quantum mechanically derived value for the rotational frequency of MA, we will take this classical value to be a point of comparison with respect to similarly classically-derived quantities of the remaining hydrated complexes. The hydrated complexes featured variable rotational behavior, correlated to the breaking and making of hydrogen bonds with the complex. These variations provide justification to cleave the full dynamic domain into relevant sub-domains. A full discussion as to the domain cleavage can be found in $\S$ SI.II.F.

Graphical representations of the dFT for each of the discussed systems is given in Figs SI25–29, one figure for each of the *n* systems. Within this representation peaks represent oscillation frequencies that appear in the relevant data, and the amplitude of the peak speaks to the relative degree that this oscillation frequency contributes to the time dynamics of the input data; data specific to cleavage regions are given in different colors, explanations of the colors are given in the individual captions. Tables SIV–VII also give reveal the results of the dFT analysis, where values in red are the most largely contributing frequencies within each region for each system. Briefly describing the vial trend within the aforementioned table, the rotational frequency decays with the addition of more waters. For the non-hydrated MA, the dominant (and sole) rotational frequencies sit around 0.00111 PHz. The rough maximal values of the rotational frequency for each of the hydrated complexes, in order of increasing *n*: 0.0008 Hz (Region I of both the 3-Cleave and 5-Cleave cases), 0.0005 PHz, 0.0003 PHz and 0.00018 PHz.

This decline in frequency with respect to additional waters speaks to a slower angular velocity in both the polar and azimuthal degrees of freedom. As it has been suggested that the rotary motion of the organic component of the lead organic perovskites is responsible for the charge carrying capacity, a decline in this motion should lead to a decline in the charge transport. As is shown through the above discussed, the addition of one water leads to a decrease in rotational velocity likely due to an increase in the moment of inertia, as it is clear that the water attempts to rotate with the MA due to the strong intermolecular forces between the molecules. This effect is increased with the addition of subsequent waters. Furthermore, the slowing of the MA will probably be greater within the lead-halide perovskite-cage, as the waters will also associate with the cage, causing an asymmetric hindered rotor effect in addition to the greater moment of inertia.

## Conclusion

Using first principles simulations, we study the solvation structure for one MA with up to four water molecules; that was done in order to understand the processes involved in the solvation and degradation and its effects on the MA’s dynamic rotation. Calculation and analysis of both the geometric and the dynamic properties have been carried out on several MA $\cdot$ (H_2_O)*n* isomers. These analysis confirmed that the nature of interaction is mostly electrostatic between the MA and the water molecule via h-bonding between the ammonium hydrogen and the oxygen of the water molecule. As the number of water molecules is increased, there is an increased chance that an isomer may form where a single water forms a hydrogen bond with another water (effectively forming a chain of hydrogen bonds leading to the ammonium); this dangling water may further stabilize the complex. The IR spectra were calculated to highlight the role of water on the dynamics of the MA, especially important concerning the vibrational modes of the amine N-H were the h-bonds are formed. Through comparative analysis of the dynamics associated with MA solvation by one or more water molecules, it may be suggested that the water molecules slow down the rotation of the MA molecule originated from the strong hydrogen bonding between the MA and the water molecules. This deceleration of MA’s rotational dynamics was further elucidated–and verified–through a Fourier analysis of MA’s polar coordinates with respect to a fixed series of axes. These results further suggest degradation of the performance of perovskite solar cells may originate from the slowing of the MA’s rotational dynamics induced by water interactions, which does in fact affect the electron-hole recombination directly impacting performance of perovskites solar cells. These findings provide support other recent computational and experimental studies of water interactions within surface perovskite environments^15,42^.

## Supplementary information

LaTeX Supplementary File

LaTeX Supplementary File

LaTeX Supplementary File

Supplementary information
