# Supplementary material for: Role of Water on the Rotational Dynamics of the Organic Methylammonium Cation: A First Principles Analysis
Source: Sci Rep. 2019 Jan 24;9:668. doi: 10.1038/s41598-018-36900-4 (PMC6346111; doi:10.1038/s41598-018-36900-4)
Supplement: Supplementary file 4 — Supplementary information [file 41598_2018_36900_MOESM4_ESM.pdf]

Role of Water on the Rotational Dynamics of the Organic Methylammonium Cation : A First Principles Analysis

Ross D. Hoehn,[1,2,a)] Joseph S. Francisco,[3] Sabre Kais,[1] and Ali Kachmar[2,b)]

[1)] *Department of Chemistry, Department of Physics and Birck Nanotechnology Center, Purdue University, West Lafayette, Indiana 47907, USA*

[2)] *Qatar Environment and Energy Research Institute, Hamad Bin Khalifa University, Qatar Foundation, P.O. Box 5825, Doha, Qatar*

[3)] *Department of Chemistry, University of Nebraska-Lincoln, Lincoln, Nebraska 68588, USA*

[a)] Electronic mail: rhoehn8701@gmail.com

[b)] Electronic mail: akachmar@hbku.edu.qa

I. STATIC CALCULATIONS

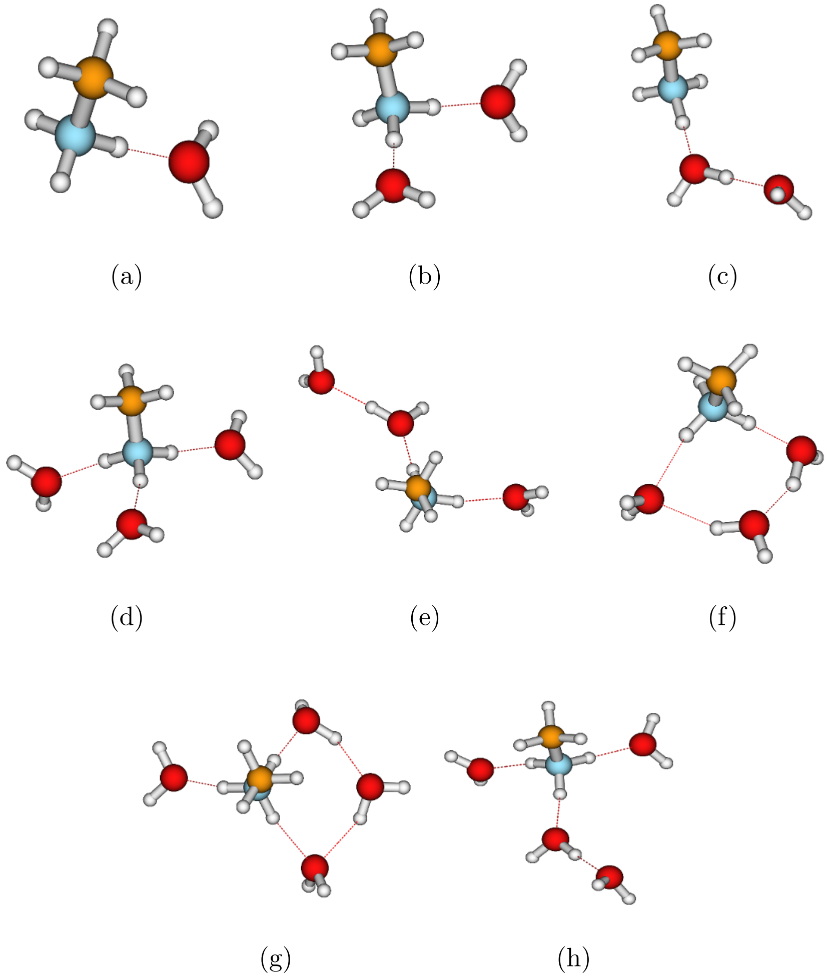

FIG. 1: Optimized structures and geometries for several MA complexes with n ($0 \geq n \leq 4$) waters. The stabilization energy (E_{stable}) of each isomer is given with respect to ($w.r.t.$) its reference geometry.

Species	Stabilization Energy (kcal/mol)	Reference Species
MA·H_2O	-19.6385	H_2O monomer
MA·$(H_2O)_2$ (Isomer 1)	-36.2049	2 H_2O monomers
MA·$(H_2O)_2$ (Isomer 2)	-29.7002	$(H_2O)_2$ dimer
MA·$(H_2O)_3$ (Isomer 1)	-50.3272	3 H_2O monomers
MA·$(H_2O)_3$ (Isomer 2)	-44.9026	$(H_2O)_2$ dimer + H_2O monomer
MA·$(H_2O)_3$ (Isomer 3)	-40.5580	$(H_2O)_3$ trimer (linear)
MA·$(H_2O)_4$ (Isomer 1)	-90.3433	$(H_2O)_2$ dimer + 2 H_2O monomers
MA·$(H_2O)_4$ (Isomer 2)	-53.6483	$(H_2O)_3$ trimer (linear) + H_2O monomer

TABLE I: Stabilization energies, E_{stable}, for each of the isomers of the hydrated complexes shown in Figure SI 1. The third column contains the reference water fragmets from which E_{stable} is calculated for each complex.

Atom	H_2O	MA	**(a)**	**(b)**	**(c)**	**(d)**	**(e)**	**(f)**	**(g)**	**(h)**
O_α	-0.385	-	-0.282	-0.280	-0.405	-0.312	-0.371	-0.314	-0.337	-0.315
O_β	-	-	-	-0.279	-0.305	-0.320	-0.411	-0.424	-0.382	-0.441
O_γ	-	-	-	-	-	-0.305	-0.302	-0.315	-0.415	-0.318
O_δ	-	-	-	-	-	-	-	-	-0.309	-0.314
$H_{\alpha 1}$	0.192	-	0.177	0.169	0.148	0.172	0.297	0.177	0.178	0.191
$H_{\alpha 2}$	0.192	-	0.177	0.174	0.327	0.175	0.123	0.175	0.181	0.190
$H_{\beta 1}$	-	-	-	0.167	0.194	0.173	0.323	0.148	0.120	0.330
$H_{\beta 2}$	-	-	-	0.166	0.193	0.169	0.127	0.321	0.291	0.146
$H_{\gamma 1}$	-	-	-	-	-	0.174	0.163	0.191	0.128	0.175
$H_{\gamma 2}$	-	-	-	-	-	0.172	0.181	0.192	0.322	0.167
$H_{\delta 1}$	-	-	-	-	-	-	-	-	0.165	0.167
$H_{\delta 2}$	-	-	-	-	-	-	-	-	0.176	0.165
N	-	0.324	0.203	0.019	0.178	-0.135	-0.031	-0.065	-0.190	-0.203
C	-	-0.431	-0.473	-0.500	-0.494	-0.520	-0.540	-0.524	-0.574	-0.554
H_{N1}	-	0.094	0.285	0.274	0.280	0.284	0.305	0.256	0.288	0.277
H_{N2}	-	0.093	0.047	0.265	0.024	0.250	0.266	0.294	0.276	0.256
H_{N3}	-	0.093	0.047	0.034	0.024	0.240	0.045	0.074	0.249	0.281
H_{C1}	-	0.277	0.275	0.280	0.278	0.270	0.252	0.282	0.249	0.266
H_{C2}	-	0.275	0.275	0.259	0.282	0.257	0.267	0.256	0.258	0.274
H_{C3}	-	0.275	0.268	0.261	0.263	0.256	0.305	0.276	0.324	0.260

TABLE II: Table containing atomistic charges (Muliken population) for all atoms within the isomers shown in Figure SI 1.

Atom	H_2O	MA	**(a)**	**(b)**	**(c)**	**(d)**	**(e)**	**(f)**	**(g)**	**(h)**
O_α	-0.922	-	-0.959	-0.949	-0.984	-0.956	-0.960	-0.961	-0.956	-0.946
O_β	-	-	-	-0.949	-0.950	-0.957	-0.980	-0.991	-0.961	-0.987
O_γ	-	-	-	-	-	-0.958	-0.970	-0.948	-0.978	-0.957
O_δ	-	-	-	-	-	-	-	-	-0.967	-0.956
$H_{\alpha 1}$	0.461	-	0.508	0.502	0.499	0.496	0.519	0.495	0.495	0.491
$H_{\alpha 2}$	0.461	-	0.508	0.502	0.529	0.496	0.497	0.498	0.495	0.493
$H_{\beta 1}$	-	-	-	0.502	0.497	0.497	0.499	0.489	0.491	0.519
$H_{\beta 2}$	-	-	-	0.502	0.497	0.496	0.495	0.522	0.517	0.486
$H_{\gamma 1}$	-	-	-	-	-	0.495	0.501	0.494	0.492	0.495
$H_{\gamma 2}$	-	-	-	-	-	0.496	0.501	0.493	0.499	0.496
$H_{\delta 1}$	-	-	-	-	-	-	-	-	0.499	0.492
$H_{\delta 2}$	-	-	-	-	-	-	-	-	0.499	0.495
N	-	-0.671	-0.697	-0.720	-0.704	-0.742	-0.723	-0.723	-0.744	-0.748
C	-	-0.389	-0.390	-0.391	-0.390	-0.390	-0.391	-0.392	-0.391	-0.389
H_{N1}	-	0.446	0.471	0.462	0.472	0.455	0.466	0.459	0.495	0.461
H_{N2}	-	0.446	0.431	0.462	0.424	0.454	0.447	0.471	0.451	0.448
H_{N3}	-	0.466	0.431	0.419	0.425	0.452	0.418	0.420	0.437	0.450
H_{C1}	-	0.240	0.234	0.227	0.230	0.223	0.230	0.227	0.216	0.221
H_{C2}	-	0.240	0.231	0.277	0.299	0.222	0.231	0.225	0.224	0.219
H_{C3}	-	0.240	0.232	0.224	0.227	0.218	0.221	0.222	0.224	0.216

TABLE III: Table containing atomistic charges (NBO poplation analysis) for all atoms within the isomers shown in Figure SI 1.

Mode	MA	H_2O	**(a)**	**(b)**	**(c)**	**(d)**	**(g)**
C-N Stretch	928.667	-	956.184	974.98	967.67	987.42	995.30
CH_3 Symmetric Bend	1430.96	-	1430.10	1428.95	1429.19	1427.27	1427.70
CH_3 Asymmetric Bend	1467.11	-	1468.39	1472.10	1469.05	1473.40	1473.38
CH_3 Asymmetric Bend	1467.25	-	1471.42	1473.15	1472.81	1476.27	1476.83
NH_3 Symmetric Bend	1499.31	-	1525.12	1552.12	1536.23	1563.58	1568.38
H_2O Bend	-	1613.02	1621.89	1621.12, 1628.50	1604.65, 1631.22	1616.55, 1623.61, 1626.04	1585.35, 1620.04, 1628.98, 1639.43
NH_3 Asymmetric Bend	1640.6	-	1646.34	1653.71	1650.59	1667.67	1672.59
NH_3 Asymmetric Bend	1640.63	-	1685.79	1689.68	1694.23	1694.27	1717.52
CH_3 Symmetric Stretch	3045.61	-	3049.33	3050.23	3049.71	3049.40	3048.76
CH_3 Asymmetric Stretch	3158.58	-	3158.08	3155.82	3157.12	3154.08	3150.83
CH_3 Asymmetric Stretch	3158.6	-	3158.66	3156.57	3157.62	3155.12	3154.15
NH_3 Symmetric Stretch	3326.13	-	3381.23	2987.95	3387.55	3085.33	-
NH_3 Asymmetric Stretch	3416.4	-	2823.16	2996.51	2495.64	3105.01	2752.71, 3135.68
NH_3 Asymmetric Stretch	3416.44	-	3442.45	3430.88	3452.1	3128.36	3270.35
H_2O Symmetric Stretch	-	3717.93	3678.49	3688.69, 3687.93	3689.3, 3193.99	3690.12, 3693.33, 3694.40	3396.31, 3583.42, 3671.26, 3691.41
H_2O Asymmetric Stretch	-	3822.39	3772.51	3783.70, 3784.14	3762.96, 3788.4	3786.35, 3790.90, 3791.25	3762.55, 3763.11, 3777.32, 3786.33

TABLE IV: Selected vibrational modes of isomers featured in Figure 1 (main text), with the addition of isomer **(c)** from Figure SI 1. The table gives the mode assignment and vibrational frequency of each vibraional mode. Frequencies colored in orange are those who are heavily coupled with neighboring modes, such that the molecular motion is a blending of the motion of these original designations. Red frequencies have become collective motions. Frequnecies in green are those which are heavily red-shifted due to the effects of hydrogen bonding; likely because the atoms committing motion within this mode directly participate in the h-bond.

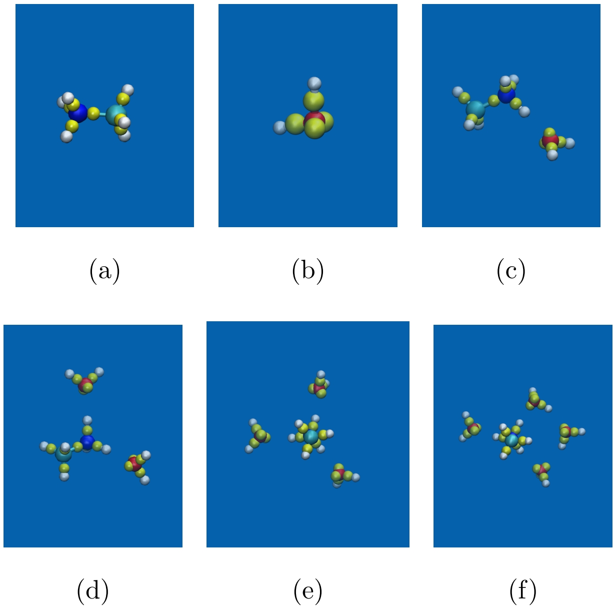

FIG. 2: Wannier centers for the optimized structure of MA, H2O and several MA$\cdot$(H_2O)$_n$ isomers. Atom colors designate: O(red), N(blue), C(green) and H(white). The centers associated with the Maximally-localized Wannier functions are represented by yellow balls. The species above (with their designations – where different – in Figure SI 1) are: MA ; H_2O ; MA$\cdot H_2O$ **(a)** ; MA$\cdot$(H_2O)$_2$ **(b)** ; MA$\cdot$(H_2O)$_3$ **(d)**; MA$\cdot$(H_2O)$_4$ **(g)**

II. DYNAMIC CALCULATIONS

A. Zero Water

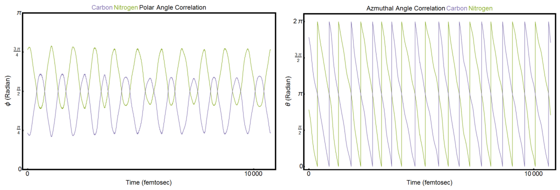

(a) (b)

FIG. 3: The dynamic polar (a) and azimuthal (b) angles for the $n = 0$ system formed by the C-N molecular axis upon a stationary coordinate system, see Figure 3 of the main text for designations.

B. One Water

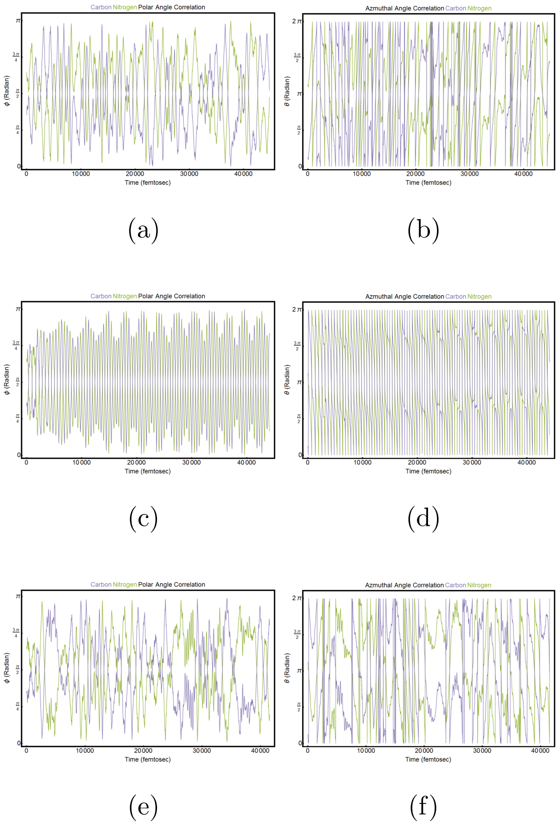

FIG. 4: The dynamic polar ((a), (c), (e)) and azimuthal ((b), (d), (f)) angles for the $n = 1$ system formed by the C-N molecular axis upon a stationary coordinate system, see Figure 3 of the main text for designations. Row 1 is for NVT simulations, row 2 for NVE simulations and row 3 for NVE-BLYP simulations.

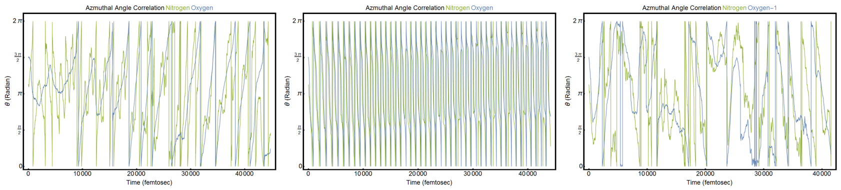

(a) (b) (c)

FIG. 5: The azimuthal angles formed by the projection of the water's oxygen upon the y-x plane, see Figure 3 of the main text for schematic representation of coordinate systems. Also shown is the dynamics azimuthal angle of nitrogen; this is done to emphasis water's ability to track the h-bonding donor. Subfigures (a)-(c) are the the NVT, NVE and NVE-BLYP simulations, respectively.

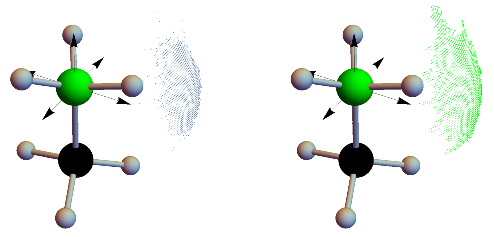

(a) (b)

FIG. 6: Cloud plots showing the distribution of h-bonds around the ammonium of MA for the NVT simulation. Such a plot for NVE and BLYP-NVE are nearly identical to this, they will not be shown. Subfigure (a) designates individual waters with different colors; as there is only one water in this case, only blue is seen. Within subfigure (b), the green distribution of particles are forming hydrogen bonds, orange region conform to distance but not angle, and red region fail both distance and angle. (Note: in this particular plot, orange and red happen to not be seen.)

C. Two Water

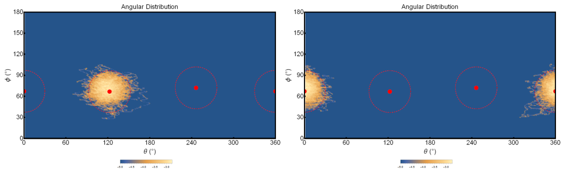

(a) (b)

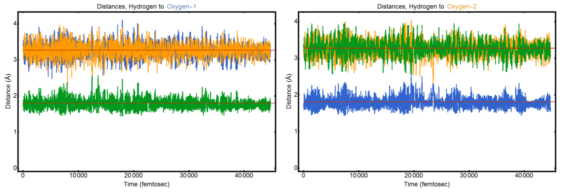

(c) (d)

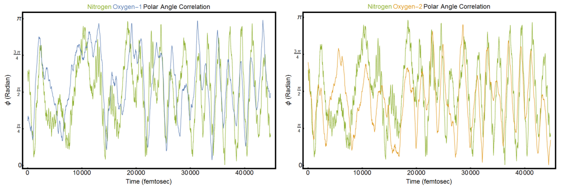

(e) (f)

FIG. 7: A collection of figures for the $n = 2$ case. Subfigures (a) and (b) are the bivariate probability plots (discussed in main text) for the individual waters. Subfigures (c) and (d) are the individual distance plots for each of the two waters; note that both waters maintain tight associations with their h-bonding partner. Subfigures (e) and (f) are the individual dyanmics of the polar coordinate for each water; also show for reference is the dynamic polar coordinate for the ammonium nitrogen.

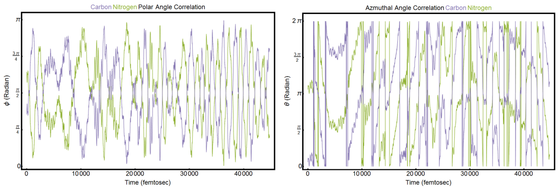

(a) (b)

FIG. 8: The dynamic polar (a) and azimuthal (b) angles for the $n = 2$ system formed by the C-N molecular axis upon a stationary coordinate system, see Figure 3 of the main text for designations.

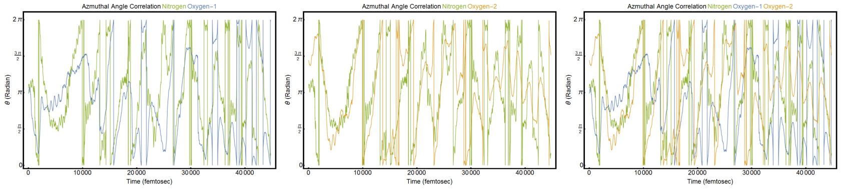

(a) (b) (c)

FIG. 9: The azimuthal angles formed by the projection of the water's oxygen upon the y-x plane, see Figure 3 of the main text for schematic representation of coordinate systems. Also shown is the dynamics azimuthal angle of nitrogen; this is done to emphasis water's ability to track the h-bonding donor. Subfigures are for individual waters.

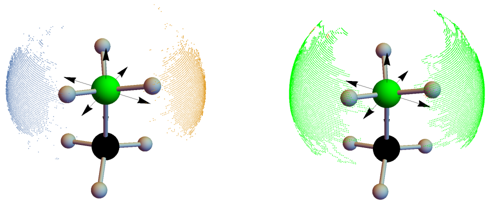

(a) (b)

FIG. 10: Cloud plots showing the distribution of h-bonds around the ammonium of MA. Such a plot for NVE and BLYP-NVE are nearly identical to this, they will not be shown. Subfigure (a) designates individual waters with different colors. Within subfigure (b), the green distribution of particles are forming hydrogen bonds, orange region conform to distance but not angle, and red region fail both distance and angle.

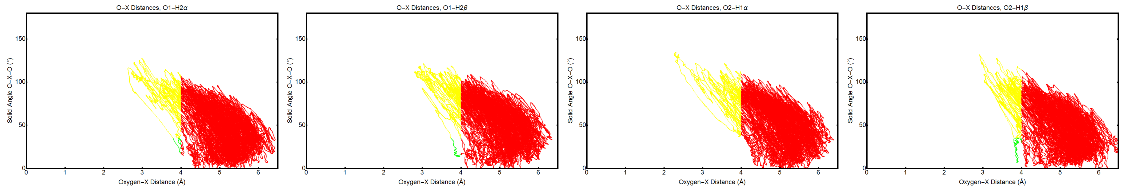

(a) (b) (c) (d)

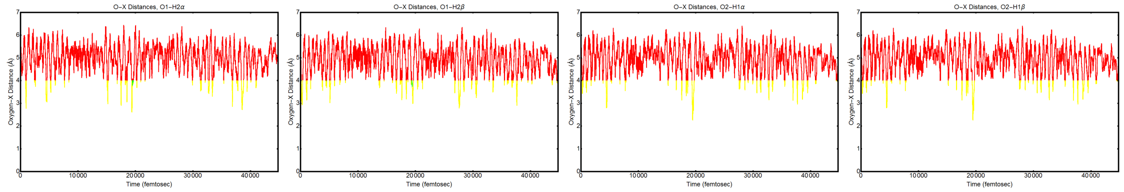

(e) (f) (g) (h)

FIG. 11: Figures conveying the water-water intermolecular interactions by examining the geometry of interactions between the individual oxygens of a water and all the hydrogens of the remaining waters. Regions in red fail both distance and angular criteria for h-bonding; yellow regions fail the angular criteria, while satisfying the distance criteria; green regions statisfy both sets of criteria. Row 1 gives a series of cluster plots where the h-bonding solid angle is plotted with respect to the h-bonding donor-acceptor distance during the MD trajectory. Row 2 plots the h-bonding donor-acceptor distance with respect to time, angle implied only through color.

D. Three Water

While Fig. SI SI 12 **(b)** reports a significantly maintained shadowing behavior for the polar coordinate (and Fig.SI 10 for the azimuthal coordinate), in agreement with the smaller n hydrated complexes, there are a few novel facets that appear in the $n = 3$ system. Fig. SI SI 12 **(a)** shows that one or more waters is more actively searching the intermolecular conformation space. In examining the individual

bivariate density plots and distance plots (Fig. SI SI 13) it is evident that there is an initial period of time (until ∼8,000fs) where a single water was not directly associated with MA, and again another water seems to walk away at ∼42,000 until the end of the simulation. The distance plots reveal a maximum distant in the range of 6Ådurring the walks, implying the formation of a dangling water in the complex; that assertion is substantiated by the bivariate distribution plot Fig. SI SI 12 **(b)** and by the h-bonding relationship between Water-2 and Water-3 shown in the analysis through Fig. SI SI 17 **(g)-(h), (k)-(l), (s)-(t)** and **(w)-(x)**. The rotational dynamics of the MA display several paradigms within the $n = 3$ complex, likely due to the formation and loss of direct associations with the MA to each water. That loss of a consistent direct association can be easily noted through the appearance of red patches in the cloud plot, Fig. SI SI 16 .

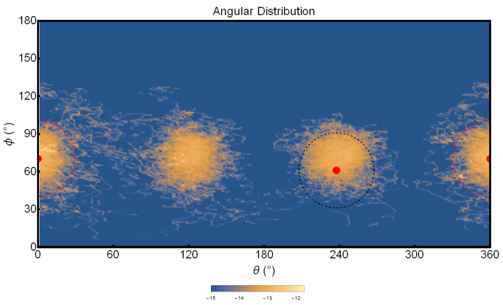

(a)

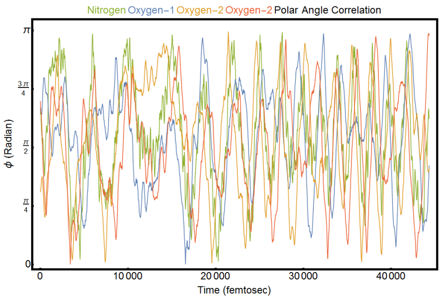

(b)

FIG. 12: Informations regarding the dynamics for the 3 water case. (a) gives the angular distributions of the water's oxygens about each of the hydrogen of MA. (b) gives the time dynamics of the polar angular coordinate.

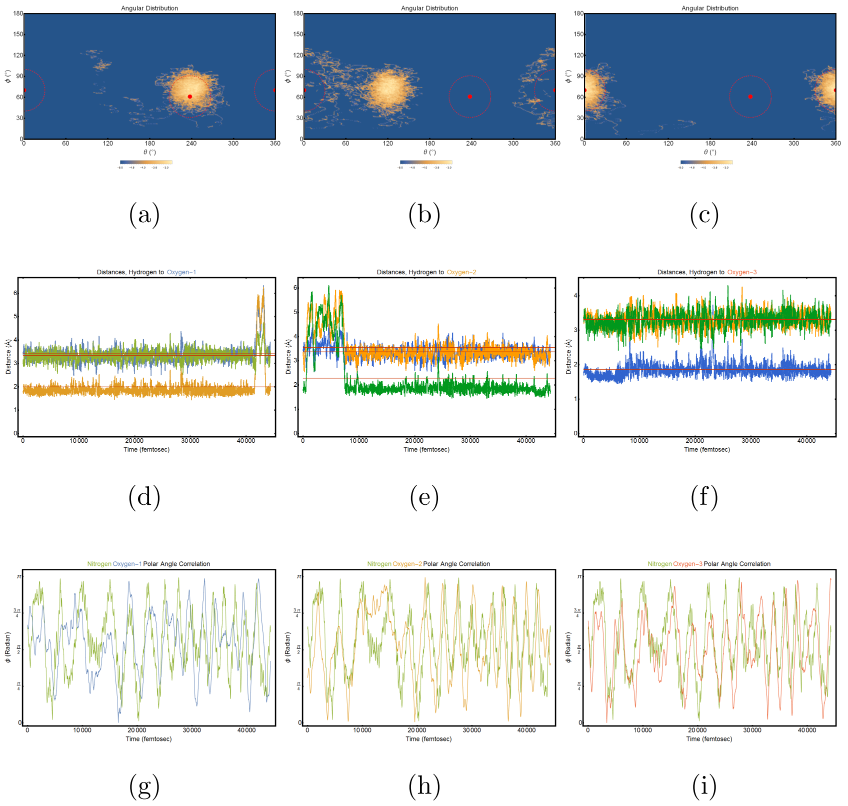

FIG. 13: A collection of figures for the $n = 3$ case. Subfigures (a)-(c) are the bivariate probability plots (discussed in main text) for the individual waters. Subfigures (d)-(f) are the individual distance plots for each of the two waters; note that both waters maintain tight associations with their h-bonding partner. Subfigures (g)-(i) are the individual dyanmics of the polar coordinate for each water; also show for reference is the dynamic polar coordinate for the ammonium nitrogen.

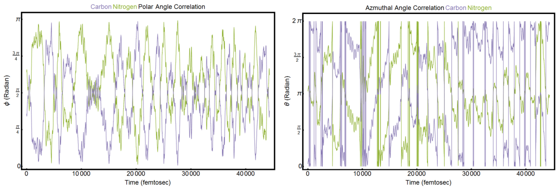

(a) (b)

FIG. 14: The dynamic polar (a) and azimuthal (b) angles for the $n = 3$ formed by the C-N molecular axis upon a stationary coordinate system, see Figure 3 of the main text for designations.

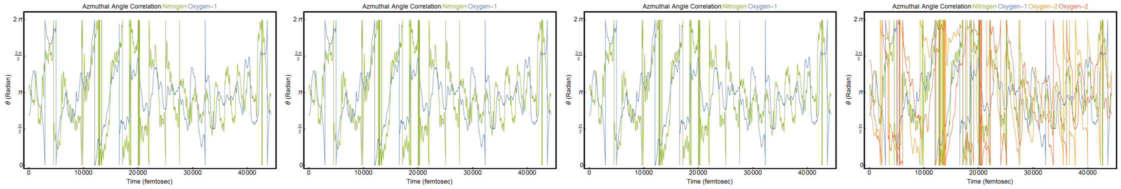

(a) (b) (c) (d)

FIG. 15: The azimuthal angles formed by the projection of the water's oxygen upon the y-x plane, see Figure 3 of the main text for schematic representation of coordinate systems. Also shown is the dynamics azimuthal angle of nitrogen; this is done to emphasis water's ability to track the h-bonding donor. Subfigures are for individual waters.

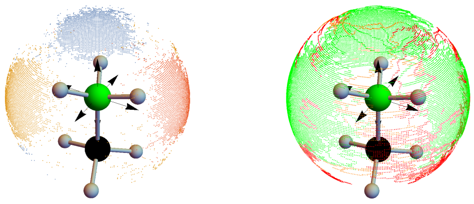

(a) (b)

FIG. 16: Cloud plots showing the distribution of h-bonds around the ammonium of MA. Such a plot for NVE and BLYP-NVE are nearly identical to this, they will not be shown. Subfigure (a) designates individual waters with different colors. Within subfigure (b), the green distribution of particles are forming hydrogen bonds, orange region conform to distance but not angle, and red region fail both distance and angle.

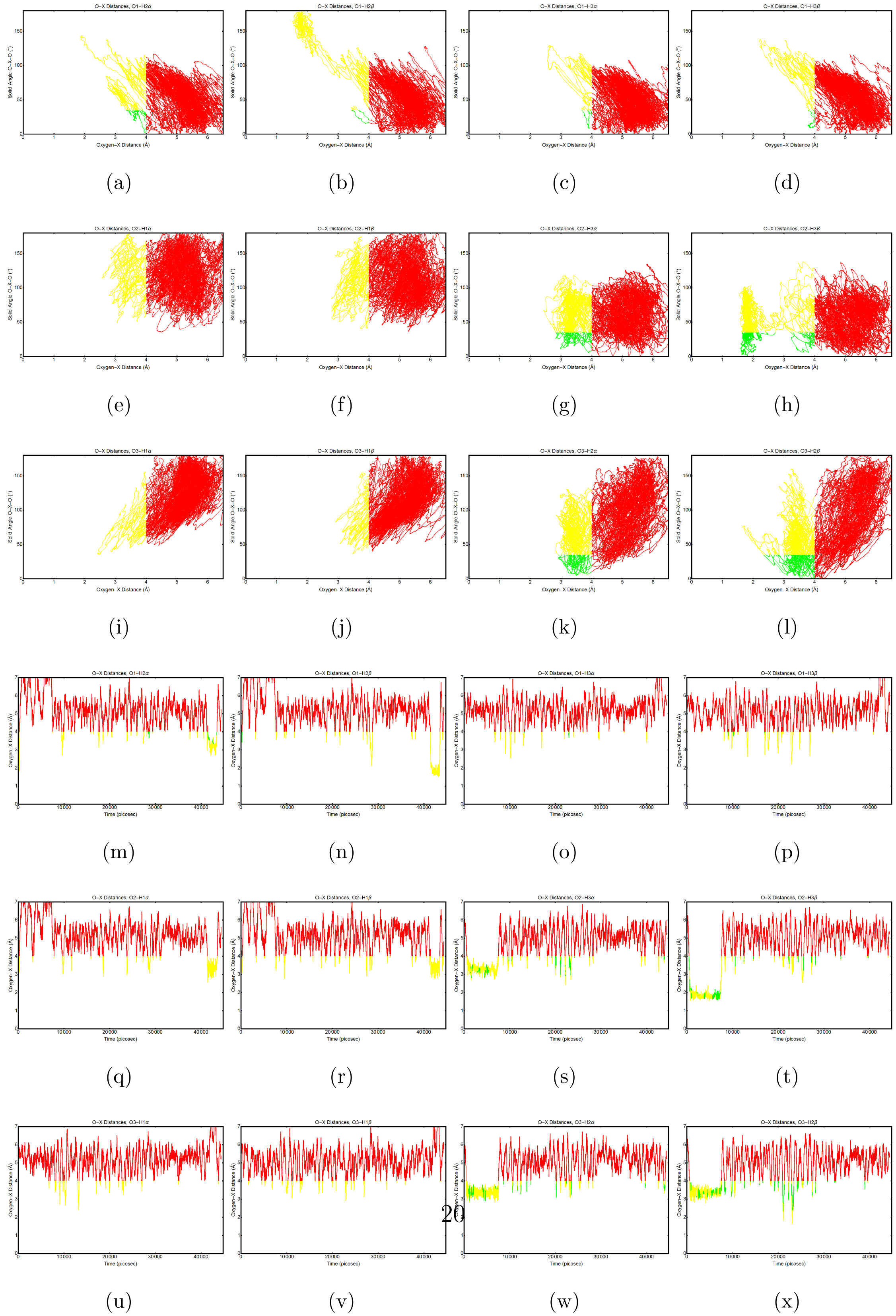

FIG. 17: Figures conveying the water-water intermolecular interactions. Regions and colors are interpretable in a similar fashion as Figure SI 11.

E. Four Water

For the $n =$ system, Fig. SI 18 **(a)** shows that there is a greater degree of conformational space exploration than the smaller systems. Another great difference is the introduction of a slight lag in the shadowing of individual waters with the ammonium group of the MA in both the polar and azimuthal coordinates, see Fig SI 18 **(b)**, Fig. SI 19 **(i)**-**(l)** and Fig. SI 21**(a)**-**(e)**. These changes in behavior are intuitive as there are now a greater number of waters than there are h-binding sites on the MA, yet it is worth noting that all waters still maintain a large degree of shadowing behavior to MA rotation. It is evident from the distance plots (Fig. SI 19 **(e)**-**(h)**) that there is a rearrangement of which waters are participating in a direct h-bond with an MA donor site; where Water-1 is initially associated with MA and Water-4 is dangling, and they switch roles. This rearrangement is evident in the regional paradigms seen in the MA rotational dynamics, see Fig. SI 20 **(a)** and **(b)**. The presence of dangling waters is evident in Figs. SI 22-SI 24 and reasonable due to the number of waters exceed the number of ammonium h-bonding donor sites.

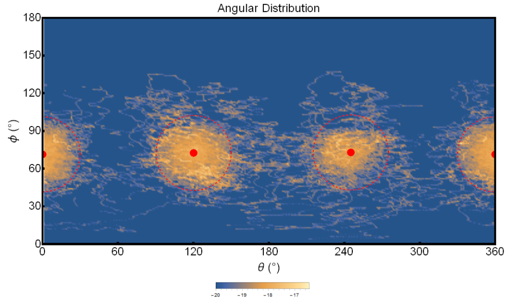

(a)

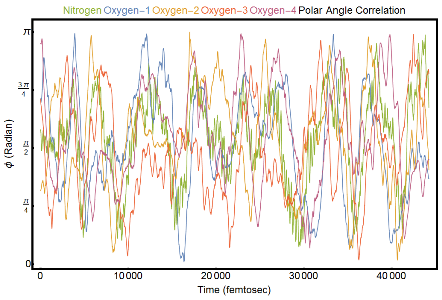

(b)

FIG. 18: Informations regarding the dynamics for the 4 water case. (a) gives the angular distributions of the water's oxygens about each of the hydrogen of MA. (b) gives the time dynamics of the polar angular coordinate.

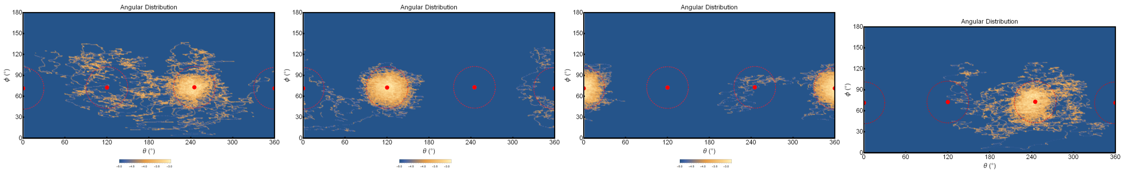

(a) (b) (c) (d)

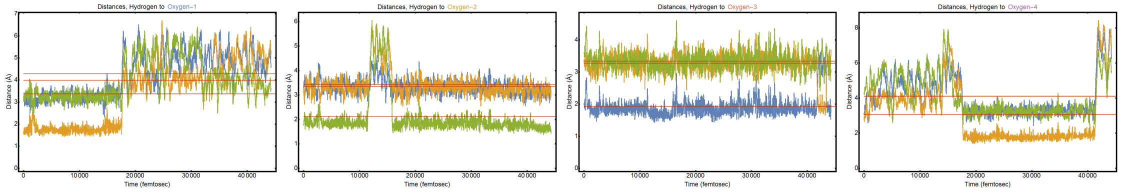

(e) (f) (g) (h)

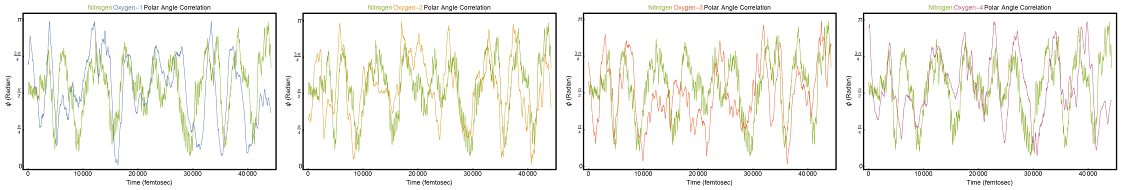

(i) (j) (k) (l)

FIG. 19: A collection of figures for the $n = 4$ case. Subfigures (a)-(d) are the bivariate probability plots (discussed in main text) for the individual waters. Subfigures (e)-(h) are the individual distance plots for each of the two waters; note that both waters maintain tight associations with their h-bonding partner. Subfigures (i)-(l) are the individual dyanmics of the polar coordinate for each water; also show for reference is the dynamic polar coordinate for the ammonium nitrogen.

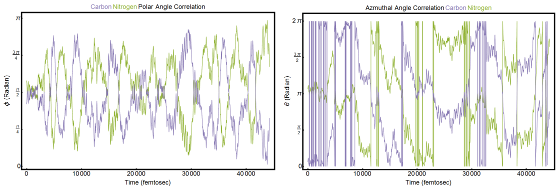

(a) (b)

FIG. 20: The dynamic polar (a) and azimuthal (b) angles for the $n = 3$ formed by the C-N molecular axis upon a stationary coordinate system, see Figure 3 of the main text for designations.

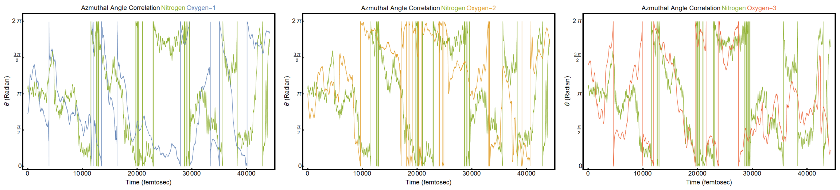

(a) (b) (c)

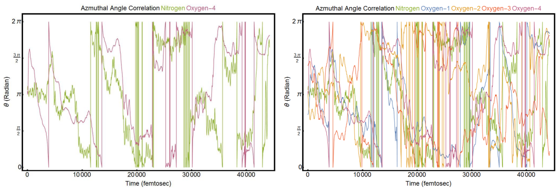

(d) (e)

FIG. 21: The azimuthal angles formed by the projection of the water's oxygen upon the y-x plane, see Figure 3 of the main text for schematic representation of coordinate systems. Also shown is the dynamics azimuthal angle of nitrogen; this is done to emphasis water's ability to track the h-bonding donor. Subfigures are for individual waters.

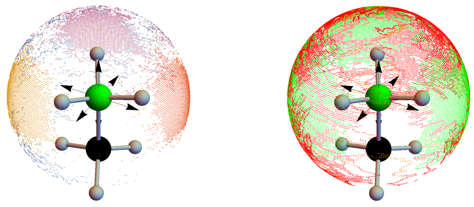

(a) (b)

FIG. 22: Cloud plots showing the distribution of h-bonds around the ammonium of MA. Such a plot for NVE and BLYP-NVE are nearly identical to this, they will not be shown. Subfigure (a) designates individual waters with different colors. Within subfigure (b), the green distribution of particles are forming hydrogen bonds, orange region conform to distance but not angle, and red region fail both distance and angle.

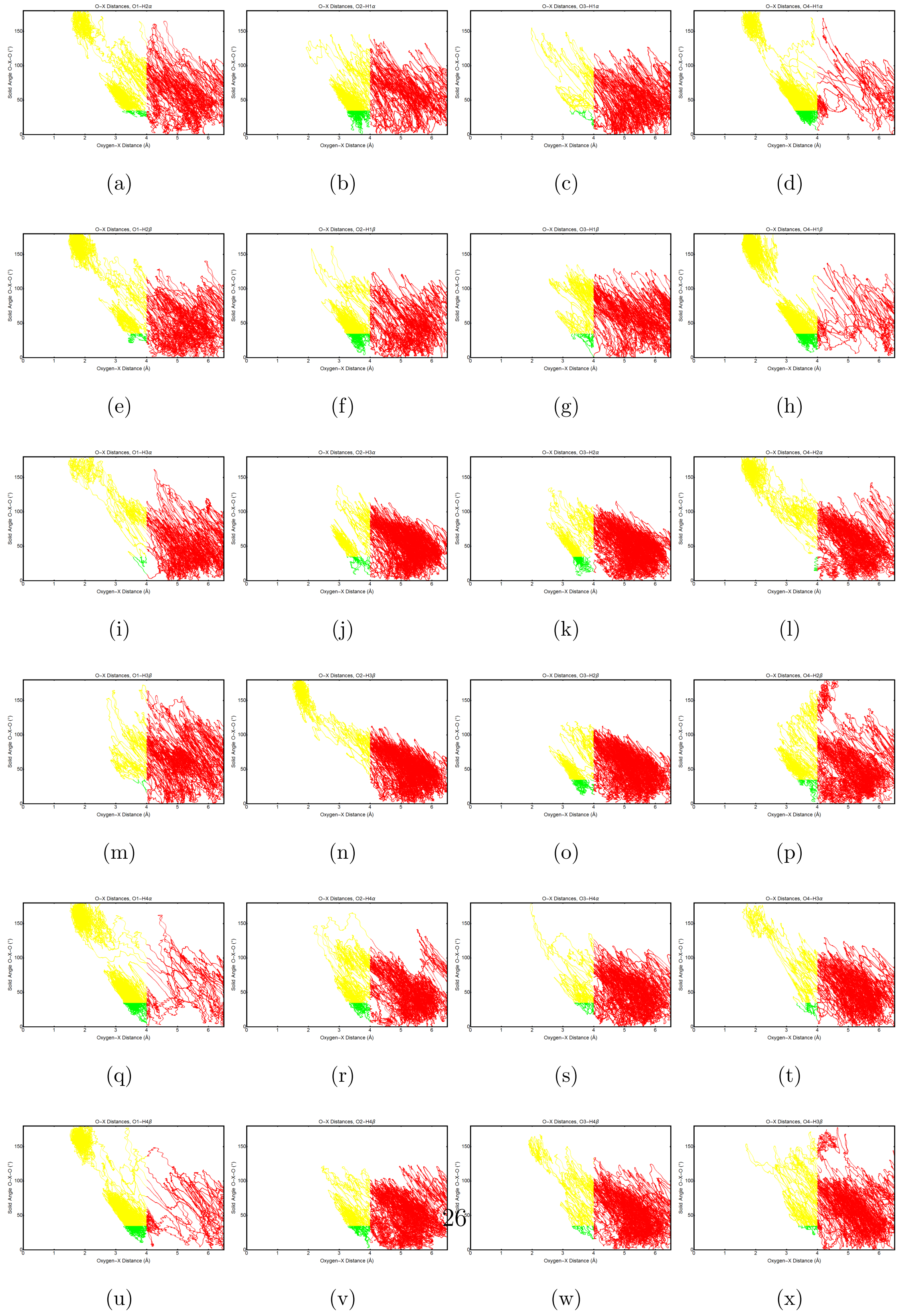

FIG. 23: Figures conveying the water-water intermolecular interactions. Regions and colors are interpretable in a similar fashion as Figure SI 11.

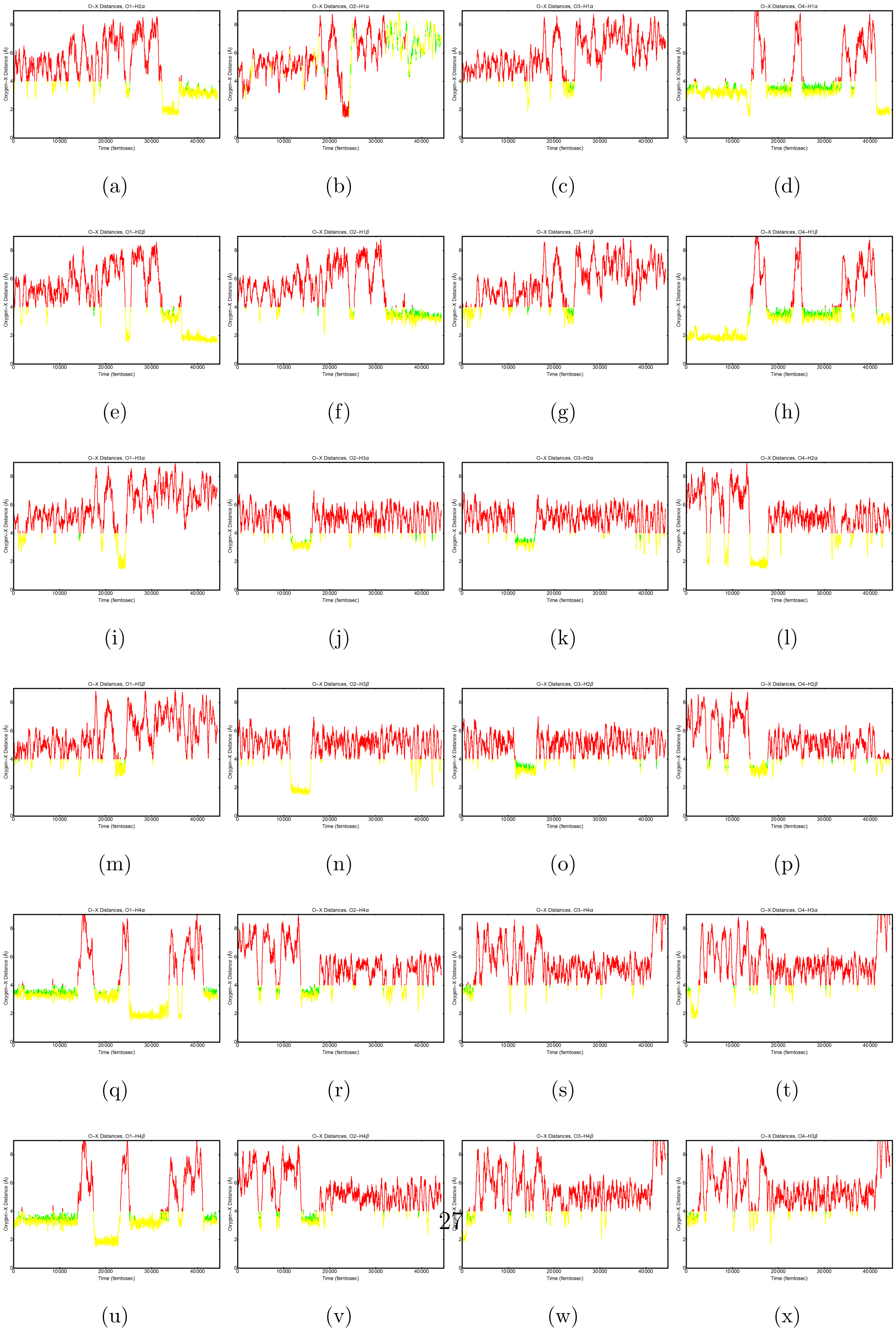

FIG. 24: Figures conveying the water-water intermolecular interactions. Regions and colors are interpretable in a similar fashion as Figure SI 11.

F. Fourier Analysis

The $n = 1$ case can be regionalized in two manners: 3-Cleave and 5-Cleave. Within each scheme, the points at which the data is cleaved are determined by behavioral aspects in the polar angular dynamics of the carbon-nitrogen bond. The cleavage points in the 3-Cleave case are: [0, 8500], (8500, 32000] and (32000, 45000], all quantities are frequency values in cm^{-1}. Similarly, those cleavage points for the case of 5-Cleavage case are: [0, 8500], (8500, 18000] , (18000, 32000], (32001, 36000] and (36000, 45000], all quantities are frequency values in cm^{-1}. The $n = 2$ case is cleaved into two regions, [0, 28000]cm^{-1} and (28000, 45000]cm^{-1}. The $n = 3$ case is cleaved into two regions, [0, 16000]cm^{-1} and (16000, 45000]cm^{-1}. A three cleavage scenario was attempted, but revealed the same significant rotational frequencies. The $n = 4$ case is cleaved into three regions based entirely on the formation of sable h-bonds (as discussed in § SI.II.D); the single region which forms the maximal number of h-bonds in a time-stable manner is the range (18000, 40500]cm^{-1}, referred to as Region II. Generally, the cleavage regions are due to specific behaviors of each system, and are direct products of the discussions in Main Paper sections MA·H_2O and MA·H_2O above and SI sections SI.II.C and SI.II.D.

coord	$n = 0$	$n = 1; 3Regions$			$n = 1; 5Regions$				
ϕ_C	0.001117	0.000840	0.000425<br>0.000563<br>0.000727<br>0.000849<br>0.001136	0.000303<br>0.000547<br>0.000946	0.000818	0.000419<br>0.000945<br>0.001263	0.000574<br>0.000786	0.000999	0.000350<br>0.000908
θ_C	0.001117	0.000824	Noise	0.000398<br>0.000626<br>0.001173	0.000824	0.000414<br>0.001263	0.000218<br>0.000504<br>0.000929	0.001258	0.000685
ϕ_N	0.001119	0.000824	0.000419<br>0.000563<br>0.000849	0.000303<br>0.000547<br>0.000945	0.000818	0.000425<br>0.000951<br>0.001270	0.000568	0.000993	0.000340<br>0.000913
θ_N	0.001097	0.000351<br>0.000829<br>0.001052	0.000425<br>0.000907<br>0.001174	0.000420<br>0.000786<br>0.001020	0.000824<br>0.001063	0.000425<br>0.001253	0.000494<br>0.000939<br>0.001147	0.000998	0.000674<br>0.001024
ϕ_{O_1}	[redacted]	None	0.000345<br>0.000425	0.000324	None	0.000425	0.000472	0.000743	0.000414
θ_{O_1}		0.000351	0.000234<br>0.000398<br>0.009713	0.000387<br>0.000855<br>0.001162	0.000351	0.000419<br>0.000748<br>0.001051	0.000287<br>0.000435<br>0.000998	0.001003	0.001008

TABLE V: Table containing frequncies present in the Fourier analysis for the 0-1 water cases. Note, there are two 1 water cases, where different schemes at cleaving the time domaine are explored. Frequencies in red text appear as strong bands within the Fourier domaine.

coord	$n = 2$		$n = 3$		$n = 4$
	0.000165				0.000175
		0.000292			
	0.000324			0.000383	0.000313
ϕ_C		0.000414	0.000319		
	0.000425			0.000515	0.000409
		0.000537			
	0.000569				0.000537
	0.000138				
	0.000228				0.000133
		0.000250	0.000190	0.000382	
θ_C	0.000452				0.000276
		0.000537	0.000313	0.000500	
	0.000568				0.000399
	0.000840				
	0.000159				0.000175
		0.000303			
	0.000329			0.000388	0.000313
ϕ_N		0.000420	0.000313		
	0.000425			0.000510	0.000404
		0.000537			
	0.000574				0.000537
	0.000106				
	0.000255				
	0.000351	0.000351	0.000186	0.000383	0.000133
θ_N	0.000537	0.000537	0.000319	0.000499	0.000271
	0.000818				
	0.0010627				

TABLE VI: Table containing frequncies present in the Fourier analysis for the 2-4 water cases for the MA molecule, we here include only domaines of interest as discussed in main text. Frequencies in red text appear as strong bands within the Fourier domaine.

coord	$n=2$		$n=3$		$n=4$
ϕ_{O_1}	0.000382<br>0.000319<br>0.000393<br>0.000574	0.000239<br>0.000531	0.000181	0.000287<br>0.000441	0.000271
θ_{O_1}	0.000202<br>0.000287<br>0.000387	0.000276<br>0.000537<br>0.000834<br>0.000951	Noise	0.000292<br>0.000441	0.000186<br>0.000319<br>0.000489<br>0.000861
ϕ_{O_2}	0.000282<br>0.000462<br>0.000616	0.000287<br>0.000531	0.000308	0.000345<br>0.000494	0.000223<br>0.000313
θ_{O_2}	0.000149<br>0.000282<br>0.000553	0.000234<br>0.000537<br>0.000723	0.000308	0.000356<br>0.000484	Noise
ϕ_{O_3}			0.000276<br>0.000680<br>0.000877	0.000138<br>0.000356<br>0.000489	0.000170<br>0.000308<br>0.000409
θ_{O_3}			0.000244<br>0.000377<br>0.000877<br>0.001132	0.000377<br>0.000484<br>0.000739	0.000223<br>0.000256<br>0.000494<br>0.000712
ϕ_{O_4}					0.000213
θ_{O_4}					0.000181<br>0.000308<br>0.000404<br>0.000542

TABLE VII: Table containing frequncies present in the Fourier analysis for the 2-4 water cases for the oxygen's of the water molecules, we here include only domaines of interest as discussed in main text. Frequencies in red text appear as strong bands within the Fourier domaine.

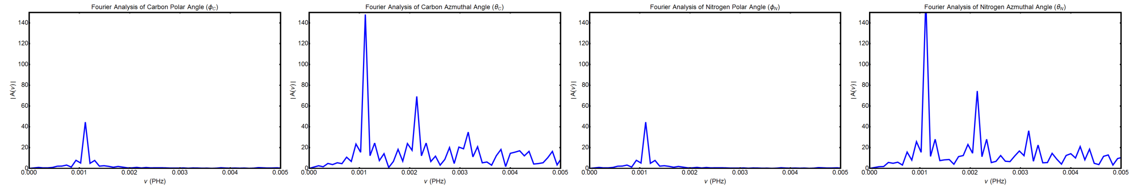

(a) (b) (c) (d)

FIG. 25: Resultant frequency analysis based on a descrete Fourier analysis of the dynamics of the Polar and Azemthal angles, individually. These plots are for the zero water case, yeilding rotational frequencies of the Polar and Azmuthal angles for the Carbon and Nitrogen of the MA.

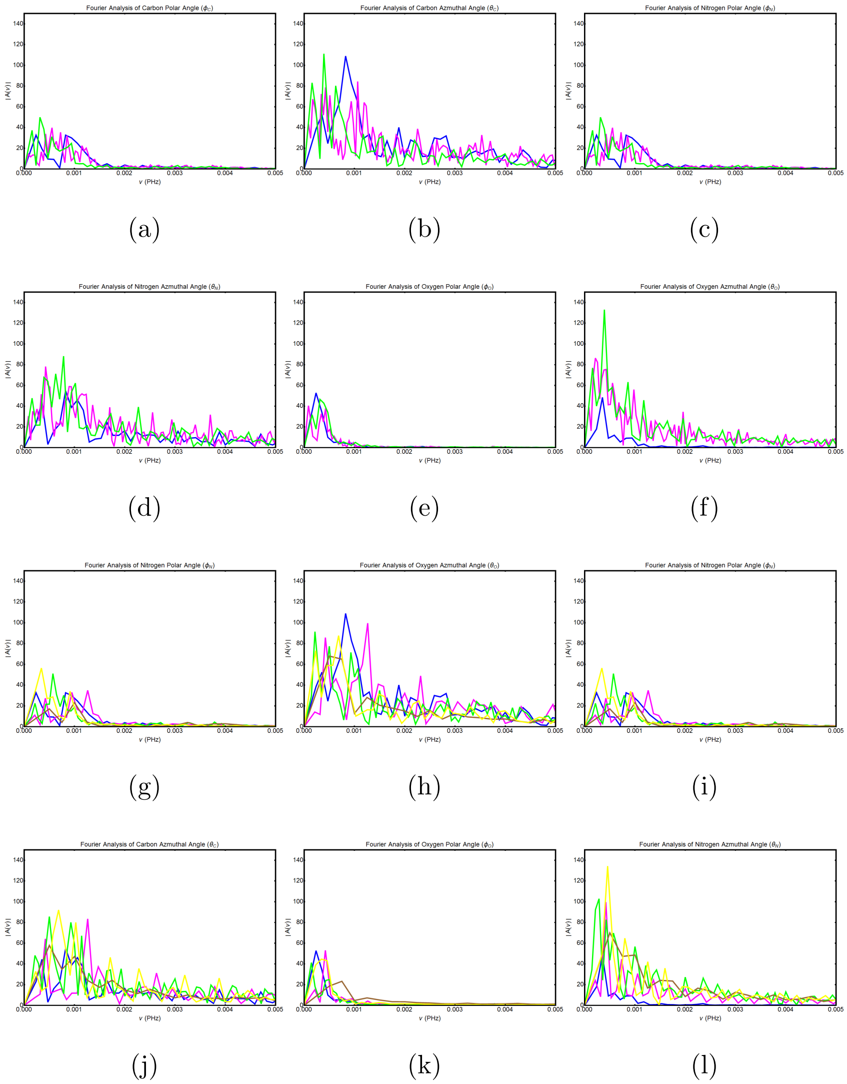

FIG. 26: Resultant frequency analysis based on a descrete Fourier analysis of the dynamics of the Polar and Azemthal angles, individually. These plots are for the one water case, yeilding rotational frequencies of the Polar and Azmuthal angles for the Carbon and Nitrogen of the MA and the Oxygen of the single water. (a)-(f) are for the 3 cleaved case, and (g)-(l) are from the 5 cleaved case.

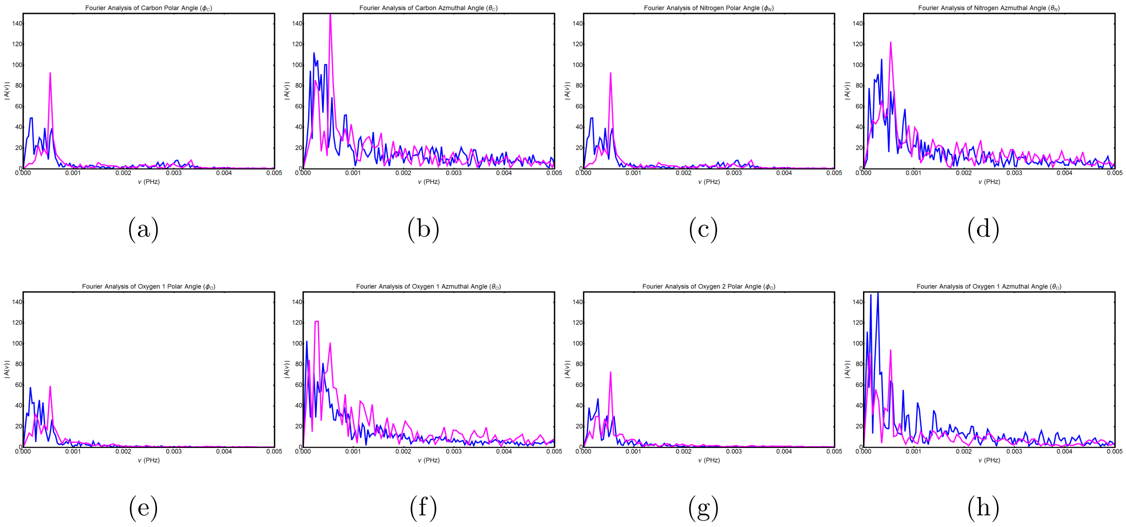

FIG. 27: Resultant frequency analysis based on a descrete Fourier analysis of the dynamics of the Polar and Azemthal angles, individually. These plots are for the two water case, yeilding rotational frequencies of the Polar and Azmuthal angles for the Carbon and Nitrogen of the MA and the Oxygens of both waters.

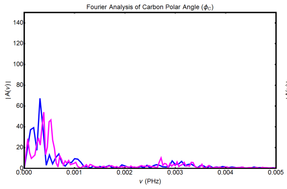

(a)

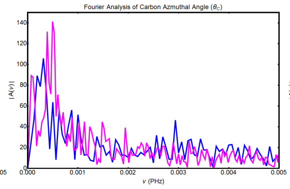

(b)

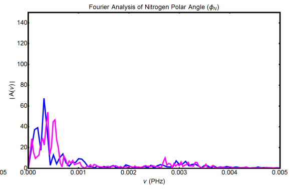

(c)

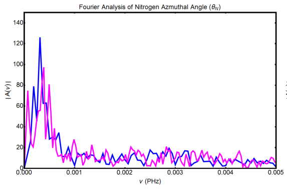

(d)

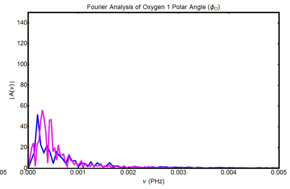

(e)

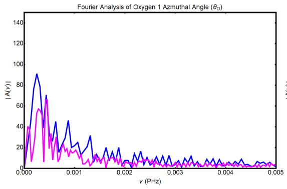

(f)

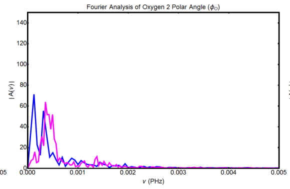

(g)

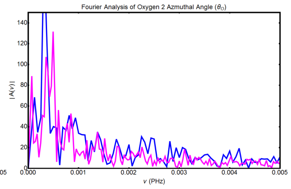

(h)

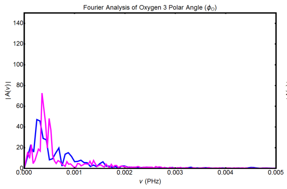

(i)

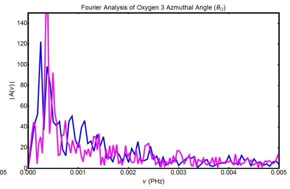

(j)

FIG. 28: Resultant frequency analysis based on a descrete Fourier analysis of the dynamics of the Polar and Azemthal angles, individually. These plots are for the three water case, yeilding rotational frequencies of the Polar and Azmuthal angles for the Carbon and Nitrogen of the MA and the Oxygens of each water in the two cleaved case.

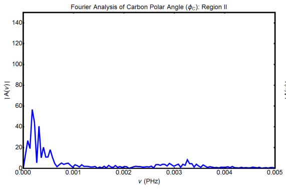

(a)

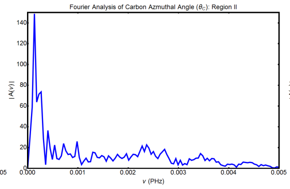

(b)

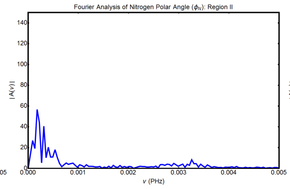

(c)

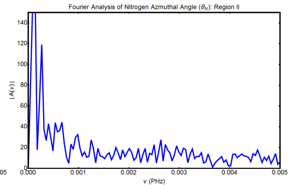

(d)

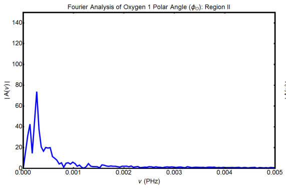

(e)

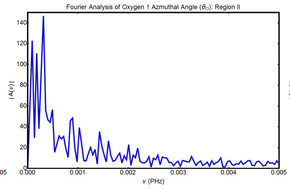

(f)

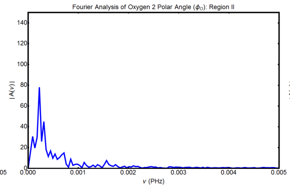

(g)

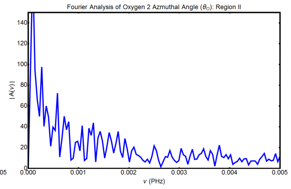

(h)

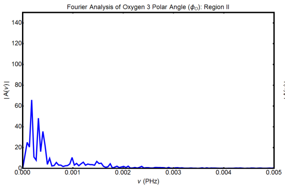

(i)

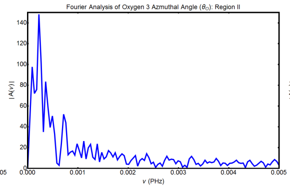

(j)

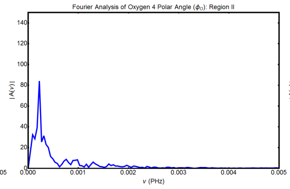

(k)

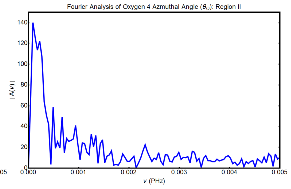

(l)

FIG. 29: Resultant frequency analysis based on a descrete Fourier analysis of the dynamics of the Polar and Azemthal angles, individually. These plots are for the four water case, yeilding rotational frequencies of the Polar and Azmuthal angles for the Carbon and Nitrogen of the MA and the Oxygens of each water.
